# TIDieR-Placebo: A guide and checklist for reporting placebo and sham controls

**DOI:** 10.1371/journal.pmed.1003294

**Published:** 2020-09-21

**Authors:** Jeremy Howick, Rebecca K. Webster, Jonathan L. Rees, Richard Turner, Helen Macdonald, Amy Price, Andrea W. M. Evers, Felicity Bishop, Gary S. Collins, Klara Bokelmann, Sally Hopewell, André Knottnerus, Sarah Lamb, Claire Madigan, Vitaly Napadow, Andrew N. Papanikitas, Tammy Hoffmann

**Affiliations:** 1 University of Oxford, Oxford, United Kingdom; 2 King’s College London, London, United Kingdom; 3 University of Oxford, Oxford, United Kingdom; 4 Public Library of Science, San Francisco, California, United States of America and Cambridge, United Kingdom; 5 The BMJ, London, United Kingdom; 6 Leiden University, Leiden, The Netherlands; 7 University of Southampton, Southampton, United Kingdom; 8 Maastricht University, Maastricht, The Netherlands; 9 Sydney University, Sydney, Australia; 10 Harvard Medical School, Cambridge, Massachusetts, United States of America; 11 Bond University, Gold Coast, Australia

## Abstract

**Background:**

Placebo or sham controls are the standard against which the benefits and harms of many active interventions are measured. Whilst the components and the method of their delivery have been shown to affect study outcomes, placebo and sham controls are rarely reported and often not matched to those of the active comparator. This can influence how beneficial or harmful the active intervention appears to be. Without adequate descriptions of placebo or sham controls, it is difficult to interpret results about the benefits and harms of active interventions within placebo-controlled trials. To overcome this problem, we developed a checklist and guide for reporting placebo or sham interventions.

**Methods and findings:**

We developed an initial list of items for the checklist by surveying experts in placebo research (n = 14). Because of the diverse contexts in which placebo or sham treatments are used in clinical research, we consulted experts in trials of drugs, surgery, physiotherapy, acupuncture, and psychological interventions. We then used a multistage online Delphi process with 53 participants to determine which items were deemed to be essential. We next convened a group of experts and stakeholders (n = 16). Our main output was a modification of the existing Template for Intervention Description and Replication (TIDieR) checklist; this allows the key features of both active interventions and placebo or sham controls to be concisely summarised by researchers. The main differences between TIDieR-Placebo and the original TIDieR are the explicit requirement to describe the setting (i.e., features of the physical environment that go beyond geographic location), the need to report whether blinding was successful (when this was measured), and the need to present the description of placebo components alongside those of the active comparator.

**Conclusions:**

We encourage TIDieR-Placebo to be used alongside TIDieR to assist the reporting of placebo or sham components and the trials in which they are used.

## Abstract 2

Jeremy Howick and colleagues recommend the use of the TIDieR-Placebo checklist for reporting of sham or placebo controls in trials.

## Background

Placebo or sham controls are the standard against which the effectiveness of many active interventions are compared [1]. To appraise whether an active intervention is effective therefore requires that the placebo or sham intervention be described. However, a decade of research shows that placebo or sham interventions in trials are rarely reported in adequate detail. A 2010 systematic review found that between 8.2% (pharmacological) and 26.7% (nonpharmacological) trials adequately described placebo or sham interventions [2]. Ten years later, things have barely improved [3]. Hence, the need to look more closely at what placebo or sham comparators are is becoming more widely recognised [4].

A possible reason for the failure to describe placebo or sham components is the misconception that they are inactive or inert [5]. There would be little point in describing inert things. In fact, placebos and sham interventions are a heterogeneous group of interventions that can cause benefits and harms. Even simple lactose pills are active for diabetics or people with lactose intolerance. The size, shape, and colour of drugs can influence the effects that are measured or perceived [6, 7]. In certain cultural contexts [8], for example, red tablets can produce a larger stimulating effect than blue ones [9], expensive tablets may have greater analgesic effects than generic cheap ones [10], and two placebos can be more effective than one [11]. More invasive placebo or sham interventions have greater effects [6, 7, 11]. Brand-name, expensive interventions (or those believed to be expensive and have a brand name) have been shown in some studies to have greater effects [12, 13]. In one study, cellulose acetate phthalate (typically used to coat pills) was reported to have activity against several sexually transmitted diseases, including herpes, in mice [14]. See S1 Text for additional examples.

Placebo interventions can also cause adverse events. For instance, some placebo pills deliberately contain ingredients to mimic a drug side effect to improve blinding [15]. For instance, in some trials of oseltamivir, the placebo contained dehydrocholic acid and dibasic calcium phosphate dihydrate. This was presumably done to mimic the bitter taste of the active intervention (oseltamivir powder) and maintain blinding. However, dehydrocholic acid can cause gastrointestinal symptoms [16]. This may have led to underestimating the gastrointestinal adverse events of oseltamivir, which was determined by comparing rates of gastrointestinal adverse events in drug with placebo groups. The term ‘active’ is used to qualify ‘placebo’ when the placebo contains an ingredient that mimics the side effect of the drug. To reduce conceptual ambiguity, we preserve the term ‘active’ to refer to nonplacebo interventions.

If placebo or sham interventions were always matched with their active comparator interventions, the problem with failure to describe placebo or sham interventions would not be as serious. This is because a trial comparing the suitably matched placebo or sham with an active intervention would reveal the incremental benefit and harm (if any) of the relevant components of the active intervention. However, a 2016 study found that 44% of control treatments were inadequately matched [17]. Worse, since placebo components are not well reported, we cannot determine precisely how common unmatching is. Without clear reporting, researchers and clinical decision-makers will not know what the effects of the placebo or sham comparator are and could subsequently overestimate or underestimate the benefits or harms of the active intervention they are testing.

The Consolidated Standards of Reporting Trials (CONSORT) statement recommends that trial interventions be adequately described. However, little detail is provided, and subsequent researchers have found that intervention reporting was poor [18]. To address intervention reporting, the Template for Intervention Description and Replication (TIDieR) checklist was developed to aid reporting of interventions and can be used in conjunction with CONSORT. However, neither CONSORT nor TIDieR explicitly mention placebo or sham controls, and our background research revealed that they require some modification to guide adequate reporting of these interventions [3].

To address this gap, a team of researchers, editors, and patient representatives developed TIDieR-Placebo. It modifies the original TIDieR so that it is directly applicable to placebo and sham interventions and is designed to be used alongside the original TIDieR. Some of these individuals were also involved in developing the original TIDieR.

## Methodology

We followed Enhancing the QUAlity and Transparency Of health Research (EQUATOR) Network guidance for developing this reporting guideline. The development of TIDieR-Placebo consisted of 5 main phases: (1) background research, (2) panel selection, (3) generation of initial checklist, (4) optimisation of the checklist, and (5) approval of the final checklist.

### Preliminary research

JH, TH, and RKW conducted preliminary research exploring the need to describe placebo and sham components adequately [2, 3, 19]. JH drafted and registered the protocol [20].

### Panel selection

To advise on the development of the checklist, we invited panel members who were identified based on a review of key authors in the field, as well as membership in the Society for Interdisciplinary Placebo Studies (SIPS). We ensured that researchers with the relevant expertise in different types of placebo or sham controls (physiotherapy, surgery acupuncture, drug), or reporting guidelines were invited. One of the panel members (AP) obtained ongoing feedback from 5 patient representatives throughout the guideline development process (see S1 Data for details).

### Generation of the initial checklist

The panel generated the initial list of items, including illustrative vignettes, through brainstorming and discussion and taking into account existing TIDieR items [21].

### Optimisation of the checklist

S2 Text has full details of the optimisation process.

#### Delphi survey

Members of the SIPS, together with an additional 24 people with expertise in placebo/sham controls or research reporting, were invited to take part in a multistage Delphi survey process. They were asked to rate items on the long list from 1 (omit) to 4 (essential) and were given the opportunity to provide full-text comments and recommend additional items. For subsequent rounds, participants were asked to rate the items generated in the long list and again had the opportunity to provide free-text comments. We planned on having 2 or 3 rounds depending on the level of agreement reached.

#### Consensus meeting and approval of final checklist

The results of the Delphi survey were reported at a 2-day consensus meeting with the panel members (supplemented by additional guideline reporting experts) on 10–11 September 2019, in Oxford, UK. Meeting attendees discussed the results of the Delphi survey and agreed on the final list of items.

To reduce the burden of ‘guideline fatigue’, the panel agreed to be conservative in choices to deviate from existing related checklists (especially TIDieR and CONSORT). For similar reasons, we decided that the extension should be concise and machine-readable. We also agreed that items that were highly ranked by Delphi survey respondents should be kept. Disagreements were resolved by discussion.

### Deviations from protocol

In the protocol, we anticipated having 12 members of the panel [20], and the final number was 14.

## Results

### TIDieR-Placebo panel

Fifteen individuals were invited to be part of the TIDieR-Placebo panel, and 14 agreed. Nine were female, and 5 were male; 9 were based in the United Kingdom, 2 in the United States, 2 in Continental Europe, and 1 in Australia.

### Generation of initial checklist

This resulted in a long list of 45 placebo or sham components for potential inclusion. There were 14 generic items (which included all the original TIDieR items) and other items useful only for specific types of sham interventions (such as ‘participant access to operation note detailing sham surgery’).

### Optimisation of the checklist

#### Delphi survey

For the first round of our web-based survey, we invited 172 participants, and 31% (n = 53) responded (see Table 1 for characteristics). Of the 45 items, 31 were ranked as essential or desirable by at least 85% of respondents, 14 were moderately ranked as essential or desirable by at least 65% of respondents, and 4 additional items were suggested in the free-text comments (S2 Text presents full details). For round 2, only those who responded to round 1 (n = 53) were invited to take part. Sixty-six percent (35 of 53) responded. After 2 rounds, 36 items were included in the draft checklist.

**Table 1:** Respondent characteristics.

Demographics	n = 53 (n)
Country of residence	
Europe	40
USA	7
Canada	1
Australia	1
New Zealand	1
Israel	1
Not answered	2
Profession (tick all that apply)	
Academic	36
Psychologist	19
Physician	8
Physiotherapist	3
Acupuncturist	2
Psychotherapist	2
Journal editor	1
Areas of practice (tick all that apply)	
Health and medical psychology	18
Clinical psychology	15
Psychosomatic medicine	12
Cognitive neuroscience	11
Primary care	8
Epidemiology	5
Anaesthesiology	4
Pain	4
Internal medicine	3
Psychiatry	3
Medical ethics and philosophy	3
Physiotherapy	3
Clinical trials	3
Rehabilitation	2
Surgery	2
Neurology	1
Behavioural neuroscience	1
Anthropology	1
Integrative medicine oncology	1
Wellbeing and healing	1
Chiropractic education	1
Number of years since award of doctoral degree	
≤10 years	27
10< and ≤20 years	13
20< and ≤30 years	6
>30 years	3
Not answered	4

#### Consensus meeting

Following the Delphi survey respondents, the placebo expert panel agreed that the original TIDieR items should be kept but that TIDieR-Placebo required 2 additions and several elaborations in order to be applicable to placebo or sham controls.

The 2 items most consistently ranked as ‘essential’ by >80% of Delphi respondents were reporting how placebo/sham components compared to those of the active intervention and measuring the success of blinding. To achieve the former, we recommend that descriptions of placebo or sham interventions are presented in a table alongside the active intervention’s description. S1 Table is a checklist that can be used to guide reporting of placebo and active interventions, and S2 Table contains descriptions of active and placebo/sham drug, surgery, psychology, acupuncture, and physiotherapy interventions.

The group discussed measuring the success of blinding extensively. Earlier versions of the CONSORT Statements recommended it [22], and later editions did not [23]. Whilst there are many cases in which lack of success of blinding does not indicate lower quality (for example, in medication cessation trials and most exercise trials), measuring the success of blinding often indicates whether the placebo or sham functioned as intended [24]. Hence, knowing whether blinding was successful can be useful. Methods for measuring the success of blinding have been developed and are currently in use. They include Bang’s ‘blinding index’ (BI) [25] and James’ BI [26]. Both ask patients to state whether they believed they received the experimental active intervention. In light of these considerations and to remain faithful to the Delphi survey responses, we agreed to add measuring the success of blinding as a TIDieR-Placebo item. We added a caveat that a measure of the success of blinding (or indeed any of the other items) is not to condemn researchers that have not done so but to encourage those who have measured the fidelity of blinding to report it. We plan to provide an outline of the case for and against measuring the success of blinding in a separate publication; see S3 Text for additional discussion of this point.

The additional explanations required to describe placebo or sham controls are all below and in S2 Table. There was an extensive discussion around whether to use the term ‘setting’ (which was recommended in the free-text comments by Delphi respondents but not used by the original TIDieR). In the meeting, it was agreed by most attendees that setting as well as location for item 7 (Where) can influence outcomes. Location is typically understood as geographic location, whereas setting can also include potentially important features of the location, such as lighting and noise. Birth setting trials were the most markedly affected, with between 68% and 85% of eligible women declining to be in trials because of preference for a particular healthcare setting [27]. In some trials, less specialised and less costly home settings are as effective as hospital settings, independently of geographic location [28, 29]. Studies have also found that factors such as noise, daylight deprivation, and light intensity may increase stress and pain level, as well as affect length of hospital stay [30–32]. Also, surgical patients might be recruited from outpatient clinics, preadmission clinics, or research clinics. Each of these settings comes with a different potential anxiety that affect the type of patient recruited as well as preoperative scores, which in turn can affect surgical outcomes. None of them are primarily related to geographic location [33].

An issue that arose at the consensus meeting, but not the Delphi survey, surrounded the ambiguity around whether certain control interventions are shams or placebos. For example, in one trial measuring methods to reduce stress amongst researchers of violence, researchers allocated participants to either group debriefings or 3 leisure sessions consisting of uplifting film viewings [34]. The film viewings were not described as sham interventions, and it is not clear whether they functioned as placebo/sham controls because taking ‘time out’ to watch a light-hearted film could, and indeed has been known to, change physiology [35]. Hence, in these cases, we recommend reporting the control according to TIDieR-Placebo. The same applies if the control is usual care or waiting list [36].

After the meeting, the report and checklist were distributed to the participants to ensure it reflected all decisions made, and this explanatory document was generated. It was then tested for face validity with 20 researchers who were conducting placebo or sham-controlled trials of different types. As a result, minor modifications were made.

### Final TIDieR-Placebo checklist

We have elaborated on items only when they require explanation over and above that already provided in the original TIDieR statement (see Table 2; downloadable version: S1 Table).

**Table 2:** TIDieR-Placebo checklist (to be used alongside TIDieR-Placebo guide).

Item	Where Located			Where Located	
	Primary paper (page or appendix number)	Other (details)		Primary paper (page or appendix number)	Other (details)
Active intervention			Placebo/sham intervention		
**1 Brief Name**					
Provide the name or a phrase that describes the intervention			Provide the name or a phrase that describes the placebo/sham intervention		
**2 Why**					
Describe any rationale, theory, or goal of the elements essential to the intervention			Describe any rationale, theory, or goal of the elements essential to the placebo/sham intervention*		
**3 What (materials)**					
Describe any physical or informational materials used in the intervention, including those provided to participants or used in intervention delivery or in training of intervention providers. Provide information on where the materials can be accessed (such as online appendix, URL)			Describe any physical or informational materials used in the placebo/sham intervention, including those provided to participants or used in intervention delivery or in training of intervention providers. Provide information on where the materials can be accessed (such as an online appendix, URL)		
**4 What (procedures)**					
Describe each of the procedures, activities, and/or processes used in the intervention, including any enabling or support activities			Describe each of the procedures, activities, and/or processes used in the placebo/sham intervention, including any enabling or support activities		
**5 Who provided**					
For each category of intervention provider (such as psychologist, nursing assistant), describe their expertise, background, and any specific training given			For each category of placebo/sham intervention provider (such as psychologist, nursing assistant), describe their expertise, background, and any specific training given		
**6 How**					
Describe the modes of delivery (such as face to face or by some other mechanism, such as internet or telephone) of the intervention and whether it was provided individually or in a group			Describe the modes of delivery (such as face to face or by some other mechanism, such as internet or telephone) of the placebo/sham intervention and whether it was provided individually or in a group		
**7 Where**					
Describe the type(s) of location(s) where the intervention occurred, including any necessary infrastructure or relevant features			Describe the type(s) of locations(s) and settings where the placebo/sham intervention occurred, including any necessary infrastructure or relevant features		
**8 When and how much**					
Describe the number of times the intervention was delivered and over what period of time, including the number of sessions, their schedule, and their duration, intensity, or dose			Describe the number of times the placebo/sham intervention was delivered and over what period of time, including the number of sessions, their schedule, and their duration, intensity, or dose. If relevant, include the duration of the pre- and postrandomisation consultations		
**9 Tailoring**					
If the intervention was planned to be personalised, titrated, or adapted, then describe what, why, when, and how			If the placebo/sham intervention was planned to be personalised, titrated, or adapted, then describe what, why, when, and how		
**10 Modifications**					
If the intervention was modified during the course of the study, describe the changes (what, why, when, and how)			If the placebo/sham intervention was modified during the course of the study, describe the changes (what, why, when, and how)		
**11 How well: planned**					
Planned: If intervention adherence or fidelity was assessed, describe how and by whom, and if any strategies were used to maintain or improve fidelity, describe them			Planned: If placebo/sham intervention adherence or fidelity was assessed, describe how and by whom, and if any strategies were used to maintain or improve fidelity, describe them		
**12 How well: actual**					
Actual: If intervention adherence or fidelity was assessed, describe the extent to which the intervention was delivered as planned			Actual: If placebo/sham intervention adherence or fidelity was assessed, describe the extent to which the intervention was delivered as planned		
**13 Measuring the success of blinding**					
Was blinding measured, and if so, how, and what were the results of such measurement?					

**1. Brief name: Provide the name or a phrase that describes the placebo/sham intervention**. Brand-name, expensive interventions have been shown in some studies to have greater effects (see above). The name also enables easy identification of the placebo or sham intervention in the report. Abbreviations or acronyms should be explained in full or short descriptive statements provided. Examples:

- ‘0.9% normal saline [37]’.
- ‘…the enhanced group’s active treatment was referred to as Singulair [38]’.

**2. Why: Describe any rationale, theory, or goal of the elements essential to the placebo/sham intervention**. The rationale for the placebo or sham chosen will usually (but not always) be to control for certain components of the active intervention. These components should be specified so that readers can appraise the extent to which this aim was achieved. Examples:

- ‘Because brand names are associated with increased perceived potency [38]’.
- ‘To control for therapist contact and support, expectations, homework tasks, etc, RT [Relaxation Techniques] participants were taught a series of relaxation techniques… [39]’.

**3. What (materials): Describe any physical or informational materials used in the placebo/sham intervention, including those provided to participants or used in intervention delivery or in training of intervention providers. Provide information on where the materials can be accessed (such as an online appendix, URL)**. Examples:

- ‘We used transcutaneous electrical nerve stimulation (TENS) equipment…For sham, instead of DD [Dense Disperse], a 40Hz adjustable (ADJ) wave was used [40]’.
- ‘…placebo (consisting of calcium phosphate, starch, cellulose, and magnesium stearate)…the placebo tablets were the same size and shape as the penicillin tablets, the tablets were not identical owing to the cost implications of overencapsulation (the placebo tablets were unmarked, and the penicillin tablets were marked) [41]’.

**4. What (procedures): Describe each of the procedures, activities, and/or processes used in the placebo/sham intervention, including any enabling or support activities**. Beyond describing standard procedures, reporting of more subtle procedures (such as nonverbal cues) [42, 43] and context [44] can influence outcomes. Examples:

- ‘Participants were encouraged to discuss their health anxiety and helpful ways of coping with it and to provide support to others randomised to the control group. The discussion forum was monitored by a clinical psychologist on a daily basis to ensure that discussions were conducted in a respectful manner [45]’.
- ‘The placebo group received a sham Kinesio Tape application, consisting of a single I-strip of the same tape applied transversely immediately above the point of maximum lumbar pain [46]’.

**5. Who provided: For each category of placebo/sham intervention provider (such as psychologist, nursing assistant), describe their expertise, background, and any specific training given**. [47] Examples:

- ‘4 physical therapists, all with a minimum of 5 years of clinical experience in outpatient orthopaedic settings [48]’.
- ‘Six acupuncturists trained in traditional Chinese medicine, licensed by the Texas State Board of Medical Examiners, were recruited through the American College of Acupuncture & Oriental Medicine (ACAOM). To ensure uniformity, all were Chinese, male, and had at least two years of clinical experience [40]’.

**6. How: Describe the modes of delivery (such as face to face or by some other mechanism, such as internet or telephone) of the placebo/sham intervention and whether it was provided individually or in a group**. Examples:

- ‘Three of the sessions were face-to-face, where the patient came into the hospital to meet with the therapist. The other five sessions were completed by telephone to increase the likelihood of attendance and reduce the stress of travel for participants [39]’.
- ‘Intervention was delivered over the phone [49]’.

**7. Where: Describe the type(s) of locations(s) and settings where the placebo/sham intervention occurred, including any necessary infrastructure or relevant features**. Both geographic location and features of the location (setting) can be important. Examples:

- ‘32 hospital sites in the UK [50]’.
- ‘Cadet Physical Therapy Clinic at the United States Military Academy or Keller Army Community Hospital at West Point, NY [48]’.

**8. When and how much: Describe the number of times the placebo/sham intervention was delivered and over what period of time, including the number of sessions, their schedule, and their duration, intensity, or dose**. If relevant, include the duration of the pre- and postrandomisation consultations. Reporting the pre- and postintervention consultation details is especially important in cases in which practitioners are not blinded after randomisation (as is often the case in sham surgery trials). In the prerandomisation consultation, equal time and the same ‘quality of care’ [51] might be spent describing the placebo compared with the experimental active intervention. One of our surgical trial experts (JLR) highlighted his experience of the ease with which postsurgical consultations or clinical contact with other treating health professionals can advertently or inadvertently unblind the patient and subsequently affect some clinical outcomes. For example, a recovery nurse might read an operation note (and learn which group the patient was in) and, not knowing the patient was in a trial, reveal to the patient which intervention they received. Examples:

- ‘…a loading dose of 1 g of tranexamic acid infused over 10 min, followed by intravenous infusion of …matching placebo (0·9% saline)…over 8 h [52]’.
- ‘For sham, instead of dense disperse wave, a 40Hz adjustable wave was used. Voltage was increased until the patient could feel it and then immediately turned off. Patients rested for 20’ with the needles retained, but without TENS stimulation [40]’.

**9. Tailoring: If the placebo/sham intervention was planned to be personalised, titrated, or adapted, then describe what, why, when, and how**. Examples:

- ‘Participants were asked if the tape was limiting lumbar movement and, if so, the tape was reapplied so that they had unrestricted range of motion [53]’.
- ‘Patients were allowed to receive placebo beyond radiographic progression as long as they continued to have clinical benefit [54]’.

**10. Modifications: If the placebo/sham intervention was modified during the course of the study, describe the changes (what, why, when, and how)**. Examples:

- ‘In some cases, the face-to-face were replaced with phone sessions if needed [55]’.
- ‘…the protocol was amended to require a time-out before the beginning of surgery [sham neurosurgery] on each side of the brain, with the coordinates confirmed by the surgeon and documented in writing by a study coordinator or other surgical team member before penetration of the brain [56]’.

**11. How well (planned): If placebo/sham intervention adherence or fidelity was assessed, describe how and by whom, and if any strategies were used to maintain or improve fidelity, describe them**. Planning to measure adherence to active as well as placebo/sham intervention could help detect whether an adverse event or unblinding may have contributed to change in adherence. Examples:

- ‘We also requested that patients complete a diary card on which the number of pills [placebo and active intervention] taken every day was recorded [57]’.
- ‘[Sham exercise] Sessions were recorded in a logbook to ensure compliance [58]’.

**12. How well (actual): If placebo/sham intervention adherence or fidelity was assessed, describe the extent to which the intervention was delivered as planned**. Examples:

- ‘Four patients did not receive the allocated injection (1 in the placebo group and 3 in the corticosteroid group) due to nonattendance (n = 2; 1%) or alternative medical advice (n = 2; 1%) [59]’.
- ‘…the proportion of patients who reported taking at least 75% of the tablets was similar in the two groups (79% in the penicillin group and 78% in the placebo group) [41]’.

**13. Measuring the success of blinding: Was blinding measured, and if so, how, and what were the results of such measurement?** Failure to demonstrate the success of blinding does not imply that the trial is invalid or lower quality. However, it can be useful to know whether blinding was successful in cases where it was measured. Examples:

- ‘As the James’ blinding indices were >0.5 and Bang’s blinding indices did not approach 1 or −1, participants were considered to have been blinded successfully on average [60]’.
- ‘Treatment allocation was correctly guessed by the outcome assessor in 53% (20/38) of cases receiving the placebo injection only, 39% (16/41) of cases receiving the placebo injection plus physiotherapy, 44% (18/41) of cases receiving the corticosteroid injection only, and 39% (15/38) of cases receiving the corticosteroid injection plus physiotherapy [59]’.

## Discussion

### Summary

To facilitate critical appraisal of the benefits and harms of the active interventions with which they are compared, placebo or sham controls need to be reported with the same rigour as experimental active interventions. Some features of placebo or sham controls require emphasis for complete reporting, such as nonverbal cues, setting, and success of blinding. Here, we report the development of TIDieR-Placebo, a reporting guideline intended to encourage the concise and accurate reporting of the nature and implementation of placebo or sham controls so as to aid in the interpretation and use of clinical research findings.

### Strengths and limitations

We followed the methodology recommended by the EQUATOR Network and believe this is the first guideline to explicitly incorporate patient perspectives. A limitation is that our survey for eliciting potential TIDieR-Placebo items asked readers to consider drug, surgery, psychological, and acupuncture, but not some other intervention subtypes such as physiotherapy, psychological, or behavioural interventions. To mitigate this, some members of the team are behavioural trial (CM) and physiotherapy trial (SL) experts. Also, our sample of survey respondents, whilst experts in placebo/sham interventions, may have been unrepresentative. Because our background research showed that placebo components are often ignored, we believed it was necessary to focus on researchers who were familiar with placebo and sham interventions. To mitigate this, we sent the manuscript to 20 trialists who user tested the manuscript and took their feedback into account.

### Conclusion and recommendations

TIDieR-Placebo is a user-friendly guide for reporting placebo and sham control interventions that can be used alongside other checklists. Its use will enhance the understanding and ability to implement and appraise placebo- and sham-controlled trials. We would encourage that it be recommended by journal editors, included by the EQUATOR Network, and used alongside other checklists, particularly CONSORT. Core members of the panel will work with EQUATOR, CONSORT, and TIDieR working groups to review and develop the guideline in the future.

## Supporting information

S1 Table TIDieR-Placebo checklist (to be used alongside TIDieR-Placebo guide). TIDieR, Template for Intervention Description and Replication.
(DOCX)

S2 Table Examples of TIDieR-Placebo with illustrations of pharmacological, surgical, psychological, acupuncture, behavioural*, and physiotherapy placebo/shams and active interventions. TIDieR, Template for Intervention Description and Replication.
(DOCX)

S1 Data Results of public and patient involvement. (DOCX)

S1 Text Additional examples in which choice of placebo/sham influenced benefits or harms of active intervention. (DOCX)

S2 Text Supporting information: Full report of Delphi consensus survey. (DOCX)

S3 Text Additional results. (DOCX)

## Abbreviations

BI: blinding index
CONSORT: Consolidated Standards of Reporting Trials
EQUATOR: Enhancing the QUAlity and Transparency Of health Research
SIPS: Society for Interdisciplinary Placebo Studies
TIDieR: Template for Intervention Description and Replication
