# Supplementary material for: TIDieR-Placebo: A guide and checklist for reporting placebo and sham controls
Source: PLoS Med. 2020 Sep 21;17(9):e1003294. doi: 10.1371/journal.pmed.1003294 (PMC7505446; doi:10.1371/journal.pmed.1003294)
Supplement: S1 Table — TIDieR, Template for Intervention Description and Replication. (DOCX) [file pmed.1003294.s001.docx]

**S1 Table. TIDieR-Placebo Checklist (to be used alongside TIDieR-Placebo guide)**

| **Item** | **Where located** | |  | **Where located** | |
| --- | --- | --- | --- | --- | --- |
|  | **Primary paper (page or appendix number)** | **Other (details)** |  | **Primary paper (page or appendix number)** | **Other (details)** |
| **Active intervention** |  |  | **Placebo/sham intervention** |  |  |
| **1 Brief Name** |  |  |  |  |  |
| Provide the name or a phrase that describes the intervention |  |  | Provide the name or a phrase that describes the placebo/sham intervention |  |  |
| **2 Why** |  |  |  |  |  |
| Describe any rationale, theory, or goal of the elements essential to the intervention |  |  | Describe any rationale, theory, or goal of the elements essential to the placebo/sham intervention* |  |  |
| **3 What (materials)** |  |  |  |  |  |
| Describe any physical or informational materials used in the intervention, including those provided to participants or used in intervention delivery or in training of intervention providers. Provide information on where the materials can be accessed (such as online appendix, URL) |  |  | Describe any physical or informational materials used in the placebo/sham intervention, including those provided to participants or used in intervention delivery or in training of intervention providers. Provide information on where the materials can be accessed (such as an online appendix, URL) |  |  |
| **4 What (procedures)** |  |  |  |  |  |
| Describe each of the procedures, activities, and/or processes used in the intervention, including any enabling or support activities |  |  | Describe each of the procedures, activities, and/or processes used in the placebo/sham intervention, including any enabling or support activities |  |  |
| **5 Who provided** |  |  |  |  |  |
| For each category of intervention provider (such as psychologist, nursing assistant), describe their expertise, background, and any specific training given |  |  | For each category of placebo/sham intervention provider (such as psychologist, nursing assistant), describe their expertise, background, and any specific training given |  |  |
| **6 How** |  |  |  |  |  |
| Describe the modes of delivery (such as face to face or by some other mechanism, such as internet or telephone) of the intervention and whether it was provided individually or in a group |  |  | Describe the modes of delivery (such as face to face or by some other mechanism, such as internet or telephone) of the placebo/sham intervention and whether it was provided individually or in a group |  |  |
| **7 Where** |  |  |  |  |  |
| Describe the type(s) of location(s) where the intervention occurred, including any necessary infrastructure or relevant features |  |  | Describe the type(s) of locations(s) and settings where the placebo/sham intervention occurred, including any necessary infrastructure or relevant features |  |  |
| **8 When and how much** |  |  |  |  |  |
| Describe the number of times the intervention was delivered and over what period of time including the number of sessions, their schedule, and their duration, intensity, or dose |  |  | Describe the number of times the placebo/sham intervention was delivered and over what period of time including the number of sessions, their schedule, and their duration, intensity, or dose. If relevant, include the duration of the pre-, and post-randomisation consultations |  |  |
| **9 Tailoring** |  |  |  |  |  |
| If the intervention was planned to be personalised, titrated or adapted, then describe what, why, when, and how |  |  | If the placebo/sham intervention was planned to be personalised, titrated or adapted, then describe what, why, when, and how |  |  |
| **10 Modifications** |  |  |  |  |  |
| If the intervention was modified during the course of the study, describe the changes (what, why, when, and how) |  |  | If the placebo/sham intervention was modified during the course of the study, describe the changes (what, why, when, and how) |  |  |
| **11 How well: planned** |  |  |  |  |  |
| Planned: If intervention adherence or fidelity was assessed, describe how and by whom, and if any strategies were used to maintain or improve fidelity, describe them |  |  | Planned: If placebo/sham intervention adherence or fidelity was assessed, describe how and by whom, and if any strategies were used to maintain or improve fidelity, describe them |  |  |
| **12 How well: actual** |  |  |  |  |  |
| Actual: If intervention adherence or fidelity was assessed, describe the extent to which the intervention was delivered as planned |  |  | Actual: If placebo/sham intervention adherence or fidelity was assessed, describe the extent to which the intervention was delivered as planned |  |  |
| **13 Measuring the success of blinding** |  |  |  | | |
| Was blinding measured, and if so: how, and what were the results of such measurement? |  |  |  | | |
