# Supplementary material for: TIDieR-Placebo: A guide and checklist for reporting placebo and sham controls
Source: PLoS Med. 2020 Sep 21;17(9):e1003294. doi: 10.1371/journal.pmed.1003294 (PMC7505446; doi:10.1371/journal.pmed.1003294)
Supplement: S2 Table — TIDieR, Template for Intervention Description and Replication. (DOCX) [file pmed.1003294.s002.docx]

**S2 Table. Examples of TIDieR-Placebo with Illustrations of Pharmacological, Surgical, Psychological, Acupuncture, Behavioural*, and Physiotherapy Placebo/Shams and Active Interventions.**

**(**In cases where the examples in the original TIDieR paper were placebo or sham controlled trials, we used them here, adding a description of the placebo or sham control.)

| **Active intervention** | **Placebo/sham intervention** |
| --- | --- |
| **1 Brief Name** |  |
| Provide the name or a phrase that describes the intervention* | Provide the name or a phrase that describes the placebo/sham intervention |
| “Single . . . dose of dexamethasone [1].”. | “0.9% normal saline[1].”. |
| “Traditional Chinese Acupuncture (TCA) [2].”. | “Sham acupuncture [2].”. |
| “…arthroscopic subacromial decompression (ASAD) [3].”. | “…investigational arthroscopy (AO) [3] |
| “Cognitive behavior therapy[4].”. | “Relaxation training[4].”. |
| “Weighing Group [5, 6].”. | “Lighten Up maintenance leaflet [5, 6].”. |
| “Three intervention groups LO:HI, HI:LO, LO:LO [7] | “PLA group [7].”. |
| “Standardized Kinesio Tape [8].”. | “Sham Kinesio Tape [8].”. |
| **2 Why** |  |
| Describe any rationale, theory, or goal of the elements essential to the intervention | Describe any rationale, theory, or goal of the elements essential to the placebo/sham intervention* |
| “An anatomical cause for subacromial pain has been proposed, whereby mechanical contact occurs between the rotator cuff tendons and the overlying acromion or bone spur that often forms at the anteroinferior margin of the acromion, narrowing the subacromial space. The narrowing makes physical contact more likely, particularly in certain positions of the arm (known as a painful arc). The condition is sometimes referred to as impingement. …surgical intervention … involves decompressing the subacromial space by removing the bone spur and any involved soft tissue arthroscopically, a procedure known as arthroscopic subacromial decompression [3].”. | “An entirely sham procedure (ie, a completely simulated surgery) can pose recruitment issues, therefore we used a placebo surgical intervention in which the accepted critical surgical element (bone and tissue removal in this instance) was omitted. For this study, the placebo intervention was investigational arthroscopy only [3].”. |
| “To our knowledge, there are no existing studies of cognitive behavior therapy (CBT) for fatigue in MS. However, three randomized controlled trial (RCTs) of CBT for chronic fatigue syndrome have reported significant improvements in fatigue, and CBT has been found to be effective in reducing depression in MS patients [9].”. | “To control for therapist contact and support, expectations, homework tasks, etc., RT participants were taught a series of relaxation techniques [9].”. |
| “We used a standardized acupuncture prescription considered effective by experts for chronic low back pain.15 This included 8 acupuncture points commonly used for chronic low back pain (Du 3, Bladder 23–bilateral, low back ashi point, Bladder 40– bilateral, Kidney 3–bilateral) on the low back and lower leg [10].”. | “We developed a simulated acupuncture technique using a tooth- pick in a needle guide tube, which was found to be a credible acupuncture treatment by acupuncture-naïve patients with back pain [10].”. |
| “Several trials have examined the effect of exercise interventions on liver fat, but their interpretation is limited by small sample sizes, lack of non-exercise control groups and heterogeneity in exercise modality and dose…….Therefore, establishing practical recommendations for exercise prescription is difficult based on the current evidence [7].”. | “The PLA intervention was designed to elicit no cardiometabolic improvements but to control for factors such as attention and participation in a lifestyle intervention [7].”. |
| **3 What (materials)** |  |
| Describe any physical or informational materials used in the intervention, including those provided to participants or used in intervention delivery or in training of intervention providers. Provide information on where the materials can be accessed (such as online appendix, URL) | Describe any physical or informational materials used in the placebo/sham intervention, including those provided to participants or used in intervention delivery or in training of intervention providers. Provide information on where the materials can be accessed (such as an online appendix, URL) |
| “We used transcutaneous electrical nerve stimulation (TENS) equipment …Labeled boxes with disposable Millennia needles were created for each arm. The depth of needles was shallower for sham acupuncture. For TCA [traditional Chinese acupuncture].”., TENS was set to emit a dense disperse (DD) wave impulse at 50Hz, dispersing at 15Hz, 20 cycles/minute [2] | “We used transcutaneous electrical nerve stimulation (TENS) equipment … For sham, instead of DD [dense disperse].”. wave, a 40Hz adjustable wave was used [2] |
| “…surgical intervention … involves decompressing the subacromial space by removing the bone spur and any involved soft tissue arthroscopically, a procedure known as arthroscopic subacromial decompression [3].”. | “…investigational arthroscopy … involves assessing the glenohumeral joint and shoulder tendons for other causes of pain [3].”. |
| “All verum acupuncture needles were single-use disposable needles (0.20– 0.25 mm diameter, 20–40 mm length, stainless steel needle; Asiamed) inserted 10–30mm deep, depending on location [11].”. | “For the sham acupuncture group, non-insertive Streitberger needles were placed on non- acupoints (SH1 and SH2) on the ulnar aspect of the more affected forearm. Similar to the local verum acupuncture arm, electrodes were attached to these needles, the electro-acupuncture device was turned on, but the electrodes were not inserted into an active port on the device. Subjects were told they ‘may or may not feel electrical sensations’ from this procedure [11].”. |
| “… low-dose oral penicillin (250 mg) [12] | “…placebo (consisting of calcium phosphate, starch, cellulose, and magnesium stearate)… the placebo tablets were the same size and shape as the penicillin tablets, the tablets were not identical owing to the cost implications of over encapsulation (the placebo tablets were unmarked, and the penicillin tablets were marked) [12].”. |
| **4 What (procedures)** |  |
| Describe each of the procedures, activities, and/or processes used in the intervention, including any enabling or support activities | Describe each of the procedures, activities, and/or processes used in the placebo/sham intervention, including any enabling or support activities |
| “Participants in HI:LO performed continuous cycling on the ergometer at an intensity of 60–70% of VO_2peak_ for two days per week, and performed and recorded an additional brisk walk at the same intensity at home one day per week. Training progressed from 30 min at 50% VO_2peak_ in week one to 45 min at 70% VO_2peak_ by the third week, totalling 90–135 min per week [13] | “Participants in the PLA group were prescribed a stretching, self-massage and fitball program. Participants received one fortnightly supervised session which involved instructions of new exercises and a 5 min cycle at very low intensity (30W) to maintain familiarity with the cycle ergometer. When combined with home-based sessions, participants in PLA undertook the sham exercise on three days per week [13] |
| “A central feature of the treatment was a self-help text of 118 pages, divided into 12 modules. Participants gradually gained access to the modules through an internet-based treatment platform. Each module was devoted to a specific theme and included homework exercises. The modules reflected the content of conventional CBT for hypochondriasis the participant had access to a therapist via a secure online contact system. The role of the therapist was mainly to provide feedback regarding all homework and to grant access to the succeeding treatment modules; however, the participant could contact the therapist at any time and expect a reply within 24 h. In addition, therapists encouraged inactive participants to continue the treatment work. During the treatment phase, participants also had access to an online discussion forum that enabled anonymous contact with other participants receiving internet based CBT [14].”. | “Participants were encouraged to discuss their health anxiety and helpful ways of coping with it, and to provide support to others randomised to the control group. The discussion forum was monitored by a clinical psychologist on a daily basis to ensure that discussions were conducted in a respectful manner [14].”. |
| “The procedure is performed with the patient under general anaesthesia. Skin incisions are made for the introduction of the arthroscope and required instruments. The procedure involves insertion of the arthroscope into the glenohumeral joint, where the joint surface is inspected along with the intraarticular portion of the long head of biceps and the joint surface of the rotator cuff tendons. Once this has been performed, the arthroscope is removed and inserted into the subacromial bursa, which lies above the rotator cuff tendons and beneath the acromion process of the scapula. In the bursa, the acromion and superior surface of the rotator cuff are assessed to ensure that the coracoacromial ligament and the AC joint remain intact. The projecting undersurface of the distal part of the acromion (spur) is resected. The intervention is considered a well-established and well-documented procedure. [3] | “The AO arm is the surgical comparison group. The procedure is performed with the patient under general anaesthesia. Patients will undergo a routine investigational arthroscopy of the glenohumeral joint and rotator cuff tendon. The operation will be performed in exactly the same manner as that in the ASAD group. Investigational arthroscopy has all the same essential operative components (and risks) of ASAD, but it does not involve surgical removal of the spur or bursal tissue or release of the coracohumeral ligament. The procedure does involve the GHJ and the subacromial bursa being inspected and irrigated. Structures can be assessed for integrity and damage. The rotator cuff can be assessed for evidence of full-thickness tears. The synovium and lining of the shoulder can be assessed for evidence of capsulitis, arthritis or frozen shoulder. The time spent in the operating theatre will be similar to that for the ASAD group. These measures provide the AO group with the characteristics necessary to provide a reasonable comparison and account for the placebo effects of surgery. [3] |
| “For TCA, TENS was set to emit a dense disperse (DD) wave impulse at 50Hz, dispersing at 15Hz, 20 cycles/minute. Voltage was increased slowly from 5V to 60V until maximal tolerance was achieved. Patients rested for 20’ with continuing TENS [2].”. | “The depth of needles was shallower for sham acupuncture… For sham, instead of DD, a 40Hz adjustable (ADJ) wave was used. Voltage was increased until the patient could feel it and then immediately turned off. Patients rested for 20’ with the needles retained, but without TENS stimulation [2].”. |
| “The experimental group received a standardised Kinesio Tape application in sitting position. Four blue I-strips were placed at 25% tension overlapping in a star shape over the point of maximum pain in the lumbar area. Strips were applied by pressing and adhering the central part before the ends [15].”. | “The placebo group received a sham Kinesio Tape application, consisting of a single I-strip of the same tape applied transversely immediately above the point of maximum lumbar pain [15].”. |
| **5 Who provided** |  |
| For each category of intervention provider (such as psychologist, nursing assistant), describe their expertise, background, and any specific training given | For each category of placebo/sham intervention provider (such as psychologist, nursing assistant), describe their expertise, background, and any specific training given |
| “The therapists conducting the internet-based CBT were four licensed psychologists with 1–4 years of clinical experience in delivering internet-based CBT [14] | “…monitored by a clinical psychologist [14] |
| “Study drug will be administered by a health professional at the study center (Day 1 and Day 14) and at patient's home (from Day 2 to Day 13) [16] | “Same as active intervention (Study drug will be administered by a health professional at the study center (Day 1 and Day 14) and at patient's home (from Day 2 to Day 13) [16] |
| **6 How** |  |
| Describe the modes of delivery (such as face to face or by some other mechanism, such as internet or telephone) of the intervention and whether it was provided individually or in a group | Describe the modes of delivery (such as face to face or by some other mechanism, such as internet or telephone) of the i placebo/sham intervention and whether it was provided individually or in a group |
| “Randomisation face to face, and intervention delivered over the phone [17] | “Randomisation face to face and materials given to participant [17] |
| “…were given an appointment with an acupuncturist… [2] | “Same as active intervention [2] |
| **7 Where** |  |
| Describe the type(s) of location(s) where the intervention occurred, including any necessary infrastructure or relevant features | Describe the type(s) of locations(s) and settings where the placebo/sham intervention occurred, including any necessary infrastructure or relevant features |
| “Women were recruited from three rural and one peri-urban antenatal clinic in Southern Malawi . . . tablets were taken under supervision at the clinic [18] | “Same as active intervention [18] |
| 20 centers of the American Lung Association Asthma Clinical Research Centers (ALAACRC) conducted the trial from December 2003 to December 2005 [19] | Same as active intervention (20 centers of the American Lung Association Asthma Clinical Research Centers (ALAACRC) conducted the trial from December 2003 to December 2005) [19]) |
| **8 When and how much** |  |
| Describe the number of times the intervention was delivered and over what period of time including the number of sessions, their schedule, and their duration, intensity, or dose | Describe the number of times the placebo/sham intervention was delivered and over what period of time including the number of sessions, their schedule, and their duration, intensity, or dose. If relevant, include the duration of the pre-, and post-randomisation consultations |
| “. . . a loading dose of 1 g of tranexamic acid infused over 10 min, followed by an intravenous infusion of 1 g over 8 h [20].”. | “… a loading dose of 1 g of tranexamic acid infused over 10 min, followed by intravenous infusion of …matching placebo (0·9% saline)…over 8 h [20].”. |
| “Two days per week and one session at home [7].”. | “…one fortnightly supervised session and 2-3 home based sessions per week [7].”. |
| **9 Tailoring** |  |
| If the intervention was planned to be personalised, titrated or adapted, then describe what, why, when, and how | If the placebo/sham intervention was planned to be personalised, titrated or adapted, then describe what, why, when, and how |
| “Treatment interruptions and dose reductions (to 40 mg and then to 20 mg) were used to manage adverse events. Patients continued the assigned trial regimen as long as they had clinical benefit, as judged by the investigator, or until they had unacceptable toxic effects. Patients were allowed to receive cabozantinib beyond radiographic progression as long as they continued to have clinical benefit [21].”. | “Treatment interruptions and dose reductions (to 40 mg and then to 20 mg) were used to manage adverse events. Patients continued the assigned trial regimen as long as they had clinical benefit, as judged by the investigator, or until they had unacceptable toxic effects. Patients were allowed to receive placebo beyond radiographic progression as long as they continued to have clinical benefit [21].”. |
| “A one page personalised, computer-tailored feedback letter, printed on the treating practitioner’s letterhead, summarised the participant’s health score and indicated behaviours for which they were and were not meeting guideline recommendations.. This letter encouraged the adoption of at least one behaviour not currently contributing to the individual’s Prudence Score. The decision as to which additional behaviour(s) to improve was the patient’s own [22].”. | “…the control group received an individualised letter and tailored information sheets about the five health protective behaviours not included in the Prudence Score (sun protection, updating tetanus vaccination, mammogram and Pap smear) [22].”. |
| **10 Modifications** |  |
| If the intervention was modified during the course of the study, describe the changes (what, why, when, and how) | If the placebo/sham intervention was modified during the course of the study, describe the changes (what, why, when, and how) |
| “In some cases, participants had an exceptionally good or bad day during the exercise test and may therefore have been allocated too heavy or too light training intensities. Whether this was the case was determined by the supervising physiotherapist, together with the participant. Exercise intensity was then adjusted accordingly in the following manner: 1) the actual workload was reduced by a maximum of 20% such that the original intensity for the 4th minute now represented a peak intensity of 80%. 2) if this was still too demanding, the number of cycles was reduced by a maximum of two. 3) if the above was still insufficient, the middle training session was made optional to allow participants a longer period of rest between sessions [23].”. | “In some cases, the face-to-face sessions were replaced with phone sessions if needed [23].”. |
| “In one individual, the postoperative CT scan showed that both catheter tips had been unintentionally placed in the same subthalamic nucleus. This occurred because the arc was rotated to the contralateral side after the first side was completed, but the previous target coordinates in the frame were not changed. The patient received a double-dose of AAV2-GAD on one side of the brain. No adverse events resulted from this surgical error, and this event was reported to all relevant institutional, study, and Federal regulatory boards. In response to this event, the protocol was amended to require a time-out before the beginning of surgery on each side of the brain, with the coordinates confirmed by the surgeon and documented in writing by a study coordinator or other surgical team member before penetration of the brain [24].”. | “…the protocol was amended to require a time-out before the beginning of surgery on each side of the brain, with the coordinates confirmed by the surgeon and documented in writing by a study coordinator or other surgical team member before penetration of the brain [24].”. |
| **11 How well: planned** |  |
| Planned: If intervention adherence or fidelity was assessed, describe how and by whom, and if any strategies were used to maintain or improve fidelity, describe them | Planned: If placebo/sham intervention adherence or fidelity was assessed, describe how and by whom, and if any strategies were used to maintain or improve fidelity, describe them |
| “To assess treatment fidelity, all face-to-face therapy sessions were audio taped and a random sample of 60 audiotapes (approximately 30% of total sessions taped) were analyzed by two independent raters using an adapted version of the Primary Care Therapy Rating Scale ([^26^](https://journals.lww.com/psychosomaticmedicine/Fulltext/2008/02000/A_Randomized_Controlled_Trial_of_Cognitive.11.aspx?casa_token=yzMM1EhadXcAAAAA:hRWPBspWB3ceLTVCSRf0XhohFDbFmjcUZwaCIpy56JQq9Z630OlUXEvfHWInyYO_KB44DPxhLv3kMFT5Y_NTTcluaQqAkJI#R26-11)). Individual items reflecting key aspects of each treatment were scored from 1 (not at all) to 4 (extensively) rather than 1 to 7 as in the original scale. High ratings indicated good adherence to the specific components of the protocols [9].”. | “To assess the face validity of the relaxation condition, participants rated at baseline how logical they found their treatment model and how confident they were that treatment would help their fatigue [9].”. |
| “Compliance with the exercise interventions was calculated as (total number of sessions attended/total number of sessions available) × 100 [13].”. | “Sessions were recorded in a logbook to ensure compliance [13].”. |
| **12 How well: actual** |  |
| Actual: If intervention adherence or fidelity was assessed, describe the extent to which the intervention was delivered as planned | Actual: If placebo/sham intervention adherence or fidelity was assessed, describe the extent to which the intervention was delivered as planned |
| “The mean (SD) number of physiotherapy sessions attended was 7.5 (1.9). Seven patients (9%) completed less than four physiotherapy sessions; the reasons included non-attendance, moving interstate, or recovery from pain. Of patients in the physiotherapy groups, 70% were compliant with their home exercise program during at least five of seven weeks [25].”. | “Four patients did not receive the allocated injection (1 in the placebo group and 3 in the corticosteroid group) due to nonattendance (n = 2; 1%) or alternative medical advice (n = 2; 1%) [25].”. |
| “A total of 214 participants (78%) reported taking at least 75% of the study tablets; the proportion of patients who reported taking at least 75% of the tablets was similar in the two groups [12].”. | “…the proportion of patients who reported taking at least 75% of the tablets was similar in the two groups (79% in the penicillin group and 78% in the placebo group) [12].”. |
| “The average number of completed modules in internet-based CBT was 9.1 (s.d. = 3.3). Six participants completed fewer than six modules and were considered non-completers [26].”. | “In the control group, the average number of postings was 16.1 (s.d. = 19.3). Thirty-nine of 41 participants in the control group posted at least one message [26].”. |
| “All participants completed the training and PLA interventions. Compliance with the exercise intervention was 94%, 90%, 96%, in HI:LO, LO:HI, LO:LO groups respectively [7].”. | “All participants completed the training and PLA interventions. Compliance with the exercise intervention was 82% in PLA group [7].”. |
| **13 Measuring the Success of Blinding** | |
| Was blinding measured, and if so: how, and what were the results of such measurement? | |
| “As the James' blinding indices were >0.5 and Bang's blinding indices did not approach 1 or −1, participants were considered to have been blinded successfully on average [27] | |
| “Treatment allocation was correctly guessed by the outcome assessor in 53% (20/38) of cases receiving the placebo injection only, 39% (16/41) of cases receiving the placebo injection plus physiotherapy, 44% (18/41) of cases receiving the corticosteroid injection only, and 39% (15/38) of cases receiving the corticosteroid injection plus physiotherapy [25] | |
| “Blinding was performed for both patients and postintervention assessors in the two surgical groups. For the other AMSR (Active Monitoring and Specialist Reassessment) group, blinding of the postintervention assessors only was appropriate. There was a nominated unblinded person at each participating site. This person was the main contact person for the patient and for the central study team in Oxford. Patients were encouraged to contact this person for queries, if they have any complications and if they wished to pursue a different treatment option. Blinding success was not measured [3].”. | |
| “The clinical psychologists performing the assessments were masked to treatment status and when summoned to clinician assessment all participants were instructed not to mention which intervention they had received. Following interviews, the assessing psychologists guessed allocation status for each participant. This was done to enable an analysis of the integrity of the masking. In two instances masking was broken as a consequence of the participants accidentally mentioning their treatment allocation status to the assessor. Fisher’s exact test, stratified by CGI–C scores to control for differential treatment effects, showed no significant association between assessors’ guess and actual treatment allocation (P50.58–0.052) [26].”. | |
| “At the end of the study we asked participants which treatment they thought they had received with three possible responses: TCA, non-traditional acupuncture, or not sure. No significant differences were observed: 52% in the TCA group and 43% in the sham thought they had received TCA (weighted kappa=0.05, p=0.23), showing successful blinding [2].”. | |

**Notes about sham interventions in behavioural trials**. In behavioural intervention research, the gold-standard trial design is less clear, particularly about the ideal comparator or control group[28, 29]. For that reason, behavioural trials have used a wide array of comparator groups in attempt to mimic placebos and the amount of attention included in pharmacological trials.

**References**

1. Gallagher TQ, Hill C, Ojha S, Ference E, Keamy DG, Williams M, et al. Perioperative dexamethasone administration and risk of bleeding following tonsillectomy in children: a randomized controlled trial. JAMA. 2012;308(12):1221-6. Epub 2012/09/27. doi: 10.1001/2012.jama.11575. PubMed PMID: 23011712.

2. Suarez-Almazor ME, Looney C, Liu Y, Cox V, Pietz K, Marcus DM, et al. A randomized controlled trial of acupuncture for osteoarthritis of the knee: effects of patient-provider communication. Arthritis Care Res (Hoboken). 2010;62(9):1229-36. doi: 10.1002/acr.20225. PubMed PMID: 20506122; PubMed Central PMCID: PMC3651275.

3. Beard DJ, Rees JL, Cook JA, Rombach I, Cooper C, Merritt N, et al. Arthroscopic subacromial decompression for subacromial shoulder pain (CSAW): a multicentre, pragmatic, parallel group, placebo-controlled, three-group, randomised surgical trial. Lancet. 2018;391(10118):329-38. Epub 2017/11/25. doi: 10.1016/S0140-6736(17)32457-1. PubMed PMID: 29169668; PubMed Central PMCID: PMCPMC5803129.

4. van Kessel K, Moss-Morris R, Willoughby E, Chalder T, Johnson MH, Robinson E. A Randomized Controlled Trial of Cognitive Behavior Therapy for Multiple Sclerosis Fatigue. Psychosomatic Medicine. 2008;70(2):205-13. doi: 10.1097/PSY.0b013e3181643065. PubMed PMID: 00006842-200802000-00011.

5. Daley A, Jolly K, Madigan C, Griffin R, Roalfe A, Lewis A, et al. A brief behavioural intervention to promote regular self-weighing to prevent weight regain after weight loss: a RCT. 2019;7:7. doi: 10.3310/phr07070.

6. Madigan CD, Jolly K, Roalfe A, Lewis AL, Webber L, Aveyard P, et al. Study protocol: the effectiveness and cost effectiveness of a brief behavioural intervention to promote regular self-weighing to prevent weight regain after weight loss: randomised controlled trial (The LIMIT Study). BMC Public Health. 2015;15:530-. doi: 10.1186/s12889-015-1869-0. PubMed PMID: 26041653.

7. Keating SE, Hackett DA, Parker HM, O’Connor HT, Gerofi JA, Sainsbury A, et al. Effect of aerobic exercise training dose on liver fat and visceral adiposity. Journal of Hepatology. 2015;63(1):174-82. doi: 10.1016/j.jhep.2015.02.022.

8. Gonzalez-Iglesias J, Fernandez-de-Las-Penas C, Cleland JA, Huijbregts P, Del Rosario Gutierrez-Vega M. Short-term effects of cervical kinesio taping on pain and cervical range of motion in patients with acute whiplash injury: a randomized clinical trial. J Orthop Sports Phys Ther. 2009;39(7):515-21. Epub 2009/07/04. doi: 10.2519/jospt.2009.3072. PubMed PMID: 19574662.

9. van Kessel K, Moss-Morris R, Willoughby E, Chalder T, Johnson MH, Robinson E. A randomized controlled trial of cognitive behavior therapy for multiple sclerosis fatigue. Psychosom Med. 2008;70(2):205-13. Epub 2008/02/08. doi: 10.1097/PSY.0b013e3181643065. PubMed PMID: 18256342.

10. Cherkin DC, Sherman KJ, Avins AL, Erro JH, Ichikawa L, Barlow WE, et al. A randomized trial comparing acupuncture, simulated acupuncture, and usual care for chronic low back pain. Arch Intern Med. 2009;169(9):858-66. doi: 10.1001/archinternmed.2009.65. PubMed PMID: 19433697.

11. Maeda Y, Kim H, Kettner N, Kim J, Cina S, Malatesta C, et al. Rewiring the primary somatosensory cortex in carpal tunnel syndrome with acupuncture. Brain : a journal of neurology. 2017;140(4):914-27. Epub 2017/03/24. doi: 10.1093/brain/awx015. PubMed PMID: 28334999; PubMed Central PMCID: PMCPMC5837382.

12. Thomas KS, Crook AM, Nunn AJ, Foster KA, Mason JM, Chalmers JR, et al. Penicillin to prevent recurrent leg cellulitis. N Engl J Med. 2013;368(18):1695-703. Epub 2013/05/03. doi: 10.1056/NEJMoa1206300. PubMed PMID: 23635049.

13. Keating SE, Hackett DA, Parker HM, O'Connor HT, Gerofi JA, Sainsbury A, et al. Effect of aerobic exercise training dose on liver fat and visceral adiposity. J Hepatol. 2015;63(1):174-82. Epub 2015/04/13. doi: 10.1016/j.jhep.2015.02.022. PubMed PMID: 25863524.

14. Hedman E, Andersson G, Andersson E, Ljotsson B, Ruck C, Asmundson GJ, et al. Internet-based cognitive-behavioural therapy for severe health anxiety: randomised controlled trial. Br J Psychiatry. 2011;198(3):230-6. Epub 2011/03/02. doi: 10.1192/bjp.bp.110.086843. PubMed PMID: 21357882.

15. Castro-Sanchez AM, Lara-Palomo IC, Mataran-Penarrocha GA, Fernandez-Sanchez M, Sanchez-Labraca N, Arroyo-Morales M. Kinesio Taping reduces disability and pain slightly in chronic non-specific low back pain: a randomised trial. J Physiother. 2012;58(2):89-95. Epub 2012/05/23. doi: 10.1016/S1836-9553(12)70088-7. PubMed PMID: 22613238.

16. Allas S, Caixas A, Poitou C, Coupaye M, Thuilleaux D, Lorenzini F, et al. AZP-531, an unacylated ghrelin analog, improves food-related behavior in patients with Prader-Willi syndrome: A randomized placebo-controlled trial. PLoS One. 2018;13(1):e0190849. Epub 2018/01/11. doi: 10.1371/journal.pone.0190849. PubMed PMID: 29320575; PubMed Central PMCID: PMCPMC5761957.

17. Madigan CD, Jolly K, Roalfe A, Lewis AL, Webber L, Aveyard P, et al. Study protocol: the effectiveness and cost effectiveness of a brief behavioural intervention to promote regular self-weighing to prevent weight regain after weight loss: randomised controlled trial (The LIMIT Study). BMC Public Health. 2015;15:530. Epub 2015/06/05. doi: 10.1186/s12889-015-1869-0. PubMed PMID: 26041653; PubMed Central PMCID: PMCPMC4453033.

18. van den Broek NR, White SA, Goodall M, Ntonya C, Kayira E, Kafulafula G, et al. The APPLe study: a randomized, community-based, placebo-controlled trial of azithromycin for the prevention of preterm birth, with meta-analysis. PLoS Med. 2009;6(12):e1000191. Epub 2009/12/04. doi: 10.1371/journal.pmed.1000191. PubMed PMID: 19956761; PubMed Central PMCID: PMCPMC2776277.

19. Wise RA, Bartlett SJ, Brown ED, Castro M, Cohen R, Holbrook JT, et al. Randomized trial of the effect of drug presentation on asthma outcomes: the American Lung Association Asthma Clinical Research Centers. The Journal of allergy and clinical immunology. 2009;124(3):436-44, 44e1-8. Epub 2009/07/28. doi: 10.1016/j.jaci.2009.05.041. PubMed PMID: 19632710; PubMed Central PMCID: PMC2948850.

20. collaborators C-t, Shakur H, Roberts I, Bautista R, Caballero J, Coats T, et al. Effects of tranexamic acid on death, vascular occlusive events, and blood transfusion in trauma patients with significant haemorrhage (CRASH-2): a randomised, placebo-controlled trial. Lancet. 2010;376(9734):23-32. Epub 2010/06/18. doi: 10.1016/S0140-6736(10)60835-5. PubMed PMID: 20554319.

21. Abou-Alfa GK, Meyer T, Cheng AL, El-Khoueiry AB, Rimassa L, Ryoo BY, et al. Cabozantinib in Patients with Advanced and Progressing Hepatocellular Carcinoma. N Engl J Med. 2018;379(1):54-63. Epub 2018/07/05. doi: 10.1056/NEJMoa1717002. PubMed PMID: 29972759.

22. Parekh S, Vandelanotte C, King D, Boyle FM. Improving diet, physical activity and other lifestyle behaviours using computer-tailored advice in general practice: a randomised controlled trial. International Journal of Behavioral Nutrition and Physical Activity. 2012;9(1):108. doi: 10.1186/1479-5868-9-108.

23. Heine M, Verschuren O, Hoogervorst EL, van Munster E, Hacking HG, Visser-Meily A, et al. Does aerobic training alleviate fatigue and improve societal participation in patients with multiple sclerosis? A randomized controlled trial. Mult Scler. 2017;23(11):1517-26. Epub 2017/05/23. doi: 10.1177/1352458517696596. PubMed PMID: 28528566; PubMed Central PMCID: PMCPMC5624301.

24. LeWitt PA, Rezai AR, Leehey MA, Ojemann SG, Flaherty AW, Eskandar EN, et al. AAV2-GAD gene therapy for advanced Parkinson's disease: a double-blind, sham-surgery controlled, randomised trial. Lancet Neurol. 2011;10(4):309-19. Epub 2011/03/23. doi: 10.1016/S1474-4422(11)70039-4. PubMed PMID: 21419704.

25. Coombes BK, Bisset L, Brooks P, Khan A, Vicenzino B. Effect of corticosteroid injection, physiotherapy, or both on clinical outcomes in patients with unilateral lateral epicondylalgia: a randomized controlled trial. JAMA. 2013;309(5):461-9. Epub 2013/02/07. doi: 10.1001/jama.2013.129. PubMed PMID: 23385272.

26. Hedman E, Andersson G, Andersson E, Ljótsson B, Rück C, Asmundson GJG, et al. Internet-based cognitive–behavioural therapy for severe health anxiety: randomised controlled trial. British Journal of Psychiatry. 2018;198(3):230-6. Epub 01/02. doi: 10.1192/bjp.bp.110.086843.

27. Choy O, Raine A, Hamilton RH. Stimulation of the Prefrontal Cortex Reduces Intentions to Commit Aggression: A Randomized, Double-Blind, Placebo-Controlled, Stratified, Parallel-Group Trial. J Neurosci. 2018;38(29):6505-12. Epub 2018/07/04. doi: 10.1523/JNEUROSCI.3317-17.2018. PubMed PMID: 29967006; PubMed Central PMCID: PMCPMC6705949.

28. Freedland KE, Mohr DC, Davidson KW, Schwartz JE. Usual and unusual care: existing practice control groups in randomized controlled trials of behavioral interventions. Psychosom Med. 2011;73(4):323-35. Epub 2011/05/04. doi: 10.1097/PSY.0b013e318218e1fb. PubMed PMID: 21536837; PubMed Central PMCID: PMCPMC3091006.

29. Mohr DC, Spring B, Freedland KE, Beckner V, Arean P, Hollon SD, et al. The selection and design of control conditions for randomized controlled trials of psychological interventions. Psychotherapy and psychosomatics. 2009;78(5):275-84. Epub 2009/07/16. doi: 10.1159/000228248. PubMed PMID: 19602916.
