# Supplementary material for: TIDieR-Placebo: A guide and checklist for reporting placebo and sham controls
Source: PLoS Med. 2020 Sep 21;17(9):e1003294. doi: 10.1371/journal.pmed.1003294 (PMC7505446; doi:10.1371/journal.pmed.1003294)
Supplement: S1 Data — (DOCX) [file pmed.1003294.s003.docx]

# **S1 Data. Results of Public and Patient Involvement**

Patient representatives were asked: What evidence-based features of placebos are important to report in trials? A survey of placebo experts and Delphi Consensus Process.

| Questions | Patient 1 | | Patient 2 | | Patient 3 | | | Patient 4 | |
| --- | --- | --- | --- | --- | --- | --- | --- | --- | --- |
|  | Before | After | Before | After | Before | After | Before | | After |
| Rationale for choice of placebo | 5 | 5 | 5 | 5 | 5 | 5 | 4 | | 4 |
| Similarities and differences to the active drug | 5 | 5 | 4 | 4 | 5 | 5 | 5 | | 5 |
| Verbal information given to patients regarding placebo intervention | 5 | 5 | 4 | 5 | 5 | 5 | 4 | | 4 |
| Non-verbal cues (e.g. study nurse makes eye contact, or sits behind computer screen) | 5 | 5 | 4 | 5 | 4 | 4 | 3 | | 3 |
| Written information (e.g. patient information leaflet) given regarding placebo intervention | 5 | 5 | 4 | 4 | 4 | 5 | 4 | | 4 |
| Duration of appointment | 5 | 5 | 4 | 4 | 3 | 3 | 4 | | 4 |
| Status of person allocating the placebo intervention (e.g. the study nurse) | 5 | 5 | 3 | 5 | 5 | 4 | 3 | | 4 |
| Location/setting of placebo intervention | 5 | 5 | 3 | 5 | 4 | 4 | 5 | | 3 |
| Status of person administering the placebo intervention (e.g. the patient) | 5 | 5 | 4 | 5 | 4 | 3 | 5 | | 3 |
| Level of blinding | 5 | 5 | 5 | 5 | 5 | 5 | 5 | | 5 |
| How blinding was attempted/achieved | 5 | 5 | 5 | 5 | 5 | 5 | 4 | | 5 |

Key: *1: not at all important 2: slightly important 3: moderately important 4: very important 5: absolutely important*

**Comments from patient representatives:**

*We can add a question to check about the person’s perception to their allocated treatment - whether they have received the regular drug or the placebo. (What is their opinion about the group, they are allotted to). Their perception to the allotted group may influence their results.*

*I feel the attitude of the patient about the disease and himself during the duration of the placebo intervention must also be included in the study which can help us see does behavioural attitude (positive vs negative) towards disease state helps in the recovery of the patient.*

*Status of person, no, but they would need methods and ethics training, Nonverbal cues could influence but it is vague and challenging to quantify so would not advise including this.*

*Allocation conditions need to be stated for example what relation does this person have to the study, they should be external without knowledge of study details and trained in ethics to do this properly other than that I believe status is an unfortunate term that is confusing and irrelevant*

*I couldn’t understand what the questionnaire means about the status of the person allocating the placebo intervention and Status of person administering the placebo intervention. The status as in socio-economic status or status of the person (coma/unconscious) during the treatment given and some other things. I was confused so I didn’t answer that question, I would like to answer it after the clarification.*
