# Supplementary material for: TIDieR-Placebo: A guide and checklist for reporting placebo and sham controls
Source: PLoS Med. 2020 Sep 21;17(9):e1003294. doi: 10.1371/journal.pmed.1003294 (PMC7505446; doi:10.1371/journal.pmed.1003294)
Supplement: S1 Text — (DOCX) [file pmed.1003294.s004.docx]

# **S1 Text. Additional Examples Where Choice of Placebo/Sham Influenced Benefits or Harms of Active Intervention**

- Olive oil was previously used in ‘placebo’ controls for cholesterol—lowering drugs before it was known that olive oil has cholesterol—lowering properties [1].
- A study of megestrol acetate as an intervention for anorexia associated with cancer showed an unexpected benefit compared with a lactose placebo in reducing gastrointestinal symptoms [2]. Because lactose intolerance is common in cancer patients [3], adverse reactions to the lactose placebo included gastrointestinal symptoms.
- A trial of motor control exercise for whiplash injury used elastic taping with the ‘wrong’ amount of tension compared with elastic taping with the ‘right’ amount of tension [4]. The trial showed no additional benefit of taping with the correct amount of tension. Another similar trial used a different kind of ‘sham’ taping (using a minimal amount of tape), and found that ‘full’ taping was effective [5].
- In surgery trials, knee arthroscopy has been defined as a sham intervention [6], as well as an ‘active’ intervention [7].

Two studies tested the effects of breathing meditation on state anxiety compared to different sham interventions. One trial found breathing meditation was more effective than audiobook listening [8], while another trial found breathing meditation was not significantly more effective than progressive muscle relaxation [9].

**References**

1. Golomb B. Paradox of placebo effect. Nature. 1995;375(6532):530. PubMed PMID: 7791863.

2. Loprinzi CL, Ellison NM, Schaid DJ, Krook JE, Athmann LM, Dose AM, et al. Controlled trial of megestrol acetate for the treatment of cancer anorexia and cachexia. J Natl Cancer Inst. 1990;82(13):1127-32. Epub 1990/07/04. PubMed PMID: 2193166.

3. Osterlund P, Ruotsalainen T, Peuhkuri K, Korpela R, Ollus A, Ikonen M, et al. Lactose intolerance associated with adjuvant 5-fluorouracil-based chemotherapy for colorectal cancer. Clin Gastroenterol Hepatol. 2004;2(8):696-703. Epub 2004/08/04. doi: S1542356504002939 [pii]. PubMed PMID: 15290663.

4. Gonzalez-Iglesias J, Fernandez-de-Las-Penas C, Cleland JA, Huijbregts P, Del Rosario Gutierrez-Vega M. Short-term effects of cervical kinesio taping on pain and cervical range of motion in patients with acute whiplash injury: a randomized clinical trial. The Journal of orthopaedic and sports physical therapy. 2009;39(7):515-21. Epub 2009/07/04. doi: 10.2519/jospt.2009.3072. PubMed PMID: 19574662.

5. Castro-Sanchez AM, Lara-Palomo IC, Mataran-Penarrocha GA, Fernandez-Sanchez M, Sanchez-Labraca N, Arroyo-Morales M. Kinesio Taping reduces disability and pain slightly in chronic non-specific low back pain: a randomised trial. Journal of physiotherapy. 2012;58(2):89-95. Epub 2012/05/23. doi: 10.1016/S1836-9553(12)70088-7. PubMed PMID: 22613238.

6. Sihvonen R, Paavola M, Malmivaara A, Itala A, Joukainen A, Nurmi H, et al. Arthroscopic partial meniscectomy versus sham surgery for a degenerative meniscal tear. N Engl J Med. 2013;369(26):2515-24. Epub 2013/12/27. doi: 10.1056/NEJMoa1305189. PubMed PMID: 24369076.

7. Moseley JB, O'Malley K, Petersen NJ, Menke TJ, Brody BA, Kuykendall DH, et al. A controlled trial of arthroscopic surgery for osteoarthritis of the knee. N Engl J Med. 2002;347(2):81-8. PubMed PMID: 12110735.

8. Zeidan F, Johnson SK, Diamond BJ, David Z, Goolkasian P. Mindfulness meditation improves cognition: evidence of brief mental training. Consciousness and cognition. 2010;19(2):597-605. Epub 2010/04/07. doi: 10.1016/j.concog.2010.03.014. PubMed PMID: 20363650.

9. Semple RJ. Does Mindfulness Meditation Enhance Attention? A Randomized Controlled Trial. Mindfulness. 2010;1(2):121-30. doi: 10.1007/s12671-010-0017-2.
