# Supplementary material for: TIDieR-Placebo: A guide and checklist for reporting placebo and sham controls
Source: PLoS Med. 2020 Sep 21;17(9):e1003294. doi: 10.1371/journal.pmed.1003294 (PMC7505446; doi:10.1371/journal.pmed.1003294)
Supplement: S2 Text — (DOCX) [file pmed.1003294.s005.docx]

# **S2 Text. Full Report of Delphi Consensus Survey**

# **Full Report of Delphi Consensus Survey**

# **Background**

The Template for Intervention Description and Replication (TIDieR) [1] is a checklist and guide to describing interventions used in clinical trials. It was developed because (active) interventions used in trials were often not described in sufficient detail to be replicated. Aside from active interventions, TIDieR mentions that trial reports should contain complete information about placebo control characteristics. However, in a recent review by our team [2] we found that of the 94 placebo-controlled trials published in the top journals in 2018, none specifically reported using TIDieR, and on average only 7.5 of the 12 TIDieR items were addressed for describing placebo controls (reporting was poorer for less well cited journals). In addition, there are some characteristics of placebo controls that are important to report yet are not required by the original TIDieR checklist. These include whether the placebo control is similar to the experimental intervention, as lack of matching can make placebos identifiable to both patients and researchers, jeopardising the blinding of the study [3].

This poor reporting of placebo control can lead to over- or under-estimation of experimental intervention benefits and harms [4], as such we do not know the extent to which placebo comparators lead to mistaken estimates of benefit or harm. While the TIDieR checklist is being used to solve the problem of poor reporting of active interventions [1], it needs to be adapted to create a reporting guideline that is specific for placebo controls.

# **Methods**

Development of the checklist follows the methodological framework for developing reporting guidelines suggested by the EQUATOR Network [5]. In collaboration with members from the original TIDieR steering committee and the CONSORT steering group, we established a TIDieR-Placebo steering committee of 13 members.

**Long list of placebo or sham components**

Our diverse group of experts were presented with vignettes for four different placebo randomised controlled trials (drug, acupuncture, surgery, behavioural/psychological), see Table A, and generated a long list of potential placebo or sham components that could be reported in study manuscripts for these trials. These consisted of 14 potential items general to all placebo randomised control trials, and an additional nine related to drug trials, four to acupuncture trials, seven to surgery trials and 11 to psychological/behavioural trials. The list was used to inform the Delphi survey.

**Table A. Placebo/sham-controlled trial vignettes**

| **Trial type** | **Vignette** |
| --- | --- |
| Drug | A randomised controlled trial testing the effect of drug X vs placebo for treating Parkinson Disease is being conducted at an outpatient clinic. At the appointment, participants complete baseline assessments and are randomised to receive drug or placebo by the study nurse. Participants leave their appointment, instructed by the study nurse to take their treatment as directed at home. Participants are followed up for 6 months. |
| Acupuncture | In a randomised controlled trial testing the effect of acupuncture vs sham acupuncture on chronic back pain, patients are referred to the study by their GP. After completing baseline assessments participants are randomised to receive four sessions of acupuncture or sham acupuncture by an acupuncturist. Follow up assessments are completed over the phone. |
| Surgery | A randomised controlled trial testing the efficacy of arthroscopic anterior cruciate ligament (ACL) reconstruction of the knee using a synthetic graft vs arthroscopic sham surgery with no graft. Patients with ACL deficient unstable knees who have failed a rehabilitation programme are recruited from hospital outpatient clinics. On the day of surgery, baseline assessments are completed by a health care practitioner (nurse or surgeon), and participants are randomised to receive the graft surgery or sham surgery. After surgery, participants are transferred to post-anesthetic recovery care and then to ward care and then home. Participants then receive extensive outpatient physiotherapy rehabilitation and are followed up at subsequent outpatient appointments. |
| Psychological/  Behavioural | A randomised controlled trial testing the efficacy of cognitive behavioural therapy (CBT) to reduce depressive symptoms in teenagers. Patients are referred to the study by their GP. After completing baseline assessments participants are randomised to receive eight sessions of CBT or ‘attention control’. After which a follow up assessment is completed over the phone. |

# **Delphi Survey**

**Design**

We used a two round modified Delphi consensus survey method [6] to identify what features of placebo controls experts think should be included in study reports.

Participants

Email invitations were sent to members of the Society for Interdisciplinary Placebo Studies (SIPS) asking them to take part, and using our teams’ extensive network, we supplemented this with others who have relevant expertise in the conduct, reporting, and implementation of clinical trials.

Procedure

Participants were presented with the four vignettes describing examples of placebo-controlled trials for drug, acupuncture, surgery and psychological/behavioural therapy (see Table A). Whilst reading these vignettes participants were asked to think about which features of the placebo control are important to report and rate these on a scale of one (omit) to four (essential), and to add their thoughts. Background information regarding participants’ country of residence, profession, areas of practice and/or research, and number of years since award of their doctoral degree was collected at the end of the survey.

From the first round, the ranked items were divided into three groups for the second round. The first group contained 31 items with the highest rankings (rated as “essential” by ≥70% participants or “essential or desirable” by ≥85%) and participants were advised that these would be included in the checklist unless strong objection to their inclusion was received in the second round. The second group contained 14 moderately ranked (rated as “essential or desirable” by ≥65% participants); participants were asked to rate each of these again as “omit,” “possible,” “desirable,” or “essential.” No items fell into the third group of lowest ranked items to be removed from the checklist (rated as “essential or desirable” by < 65% participants). Open ended responses were qualitatively synthesised and responded to in Round 2, and an additional four items suggested by participants were included in the second round. Again, participants could add comments through the provision of a free text responses.

# **Results**

**Participant characteristics**

Our sample contained 53 participants. The majority were from Europe and the USA, had an academic or Psychologist profession, with areas of research/practice concerning Health and Medical Psychology, Clinical Psychology and Psychosomatic Medicine. The average number of years since award of doctoral degree was 12.35 years. The response rate for round two was 66% (35 of 53), for round one this was 31% (53 of 172). For full sample characteristics see Table 1 (main text).

**Rankings**

After the two Delphi rounds, 36 items were included in the draft checklist, one item was excluded, and 12 moderately ranked items were retained for further discussion at the in-person meeting. For full results see Table D.

Of those 36, 11 of the items were general to all types of placebo-controlled trials and consisted of many features already listed in the original TIDieR, e.g. ‘why’, ‘what’, ‘when and how much’, ‘modifications’, ‘tailoring’ and ‘how well’. Others that were not part of the original TIDieR included level of binding, and similarities and differences between the placebo and active intervention. For the remaining 25 items specific to different types of placebo-controlled trials many of these also related to the original TIDieR items above but were specific to the type of placebo-controlled trial, e.g. ingredients, dose and route of administration of drug placebos, type of acupuncture for sham acupuncture, content of attention control for psychological/behavioural placebos. Other items that were not part of the original TIDieR included specific features related to blinding, e.g. assessment of needle sensations for sham acupuncture, and patient access to operation note for sham surgery.

Of the 12 items moderately ranked, six were general to all types of placebos and concerned more contextual factors, such as: non-verbal cues, location of placebo treatment, duration of initial consultation, how the placebo was marketed to those allocating/delivering the placebo, and participants and practitioners’ expectations. Four were specific to drug placebos and concerned the status of those who administered the placebo, size, taste and manufacturer. The remaining two concerned sham surgery which was staff access to operation notes, and training of the practitioner, which was interestingly deemed less important than for acupuncture, and psychological/behavioural placebos.

The one item which received the lowest ranking was for drug placebos concerning the status of the person allocating the placebo treatment. These items have been condensed into those that can be included in the original TIDieR (see Table B) and those which represent additional items not currently included in TIDieR (see Table C).

**Table B. Items included in the original TIDieR**

| **Item** | **Essential/desirable ≥ 85% (y), Essential/desirable ≥65 <85% (?), Essential/desirable <65% (n)** |
| --- | --- |
| Brief name | - |
| Why | Y (87.3%) |
| What (materials) | ? (91.3% average) – but manufacturer (65.7%), size (80%), taste (80%) moderately rated for drug trials |
| What (procedures) | Y (96.1% average) |
| Who provided | ? drug (administering 82.9%, allocated 57.2%), acupuncture (91.5%), surgery (82.9%), psychological/behavioural (99.2%) |
| How | Y (96.7% average) |
| Where | ? (71.4%) |
| When and how much | Y (93.9% average) |
| Tailoring | Y (88.6%) |
| Modifications | Y (85.0%) |
| How well (planned) | Y (88.2%) |
| How well (actual) | Y (89.7%) |

**Table C. Additional items not included in TIDieR**

| **Item** | **Essential/desirable ≥ 85% (y), Essential/desirable ≥65 <85% (?), Essential/desirable <65% (n)** |
| --- | --- |
| General |  |
| Level of blinding | Y (97.2%) |
| How blinding was attempted/achieved | Y (96.7%) |
| Similarities and differences to active intervention | Y (87.3%) |
| Non-verbal cues | ? (65.8%) |
| Duration of consultation prior to placebo treatment | ? (68.6%) |
| Participant/practitioner treatment expectations | ? (68.5%) |
| Marketing of placebo to those allocating/delivering treatments | ? (68.6%) |
| Acupuncture |  |
| Assessment of sham acupuncture needle sensations | Y (86.8%) |
| When did unblinding occur | Y (88.7%) |
| Surgery |  |
| Similarities and differences to ‘real’ post-surgery care/rehab | Y (96.3%) |
| Participant access to operation note detailing surgery | Y (88.7%) |
| Staff access to operation note detailing surgery | ? (68.6%) |

**Round one - open ended comments**

From round one, some of the comments requested that we add items that already exist. For example, some requested that we add ‘duration’, when ‘how long?’ is a TIDieR item. As an additional suggestion, many people promoted the need to measure patient and practitioner expectations. This could be a separate item, or it might be subsumed under blinding success. If expectations are different, then there will be no blinding. On the other hand, blinding is difficult to achieve in many interventions, so expectations might be better to measure than blinding. As such we included it as an additional item in round two. For a summary of the open comments and author responses from round one, see Tables E.

**Round two – open ended comments**

From round two there was some concern communicated about having long checklists for each type of placebo-controlled trial. It was recognised that although it would be ideal to report all of these features, in practice this is not feasible and perhaps most items could be collapsed into similarities vs differences where authors could highlight when these placebo features differ to that of the active intervention. There was also some suggestion that patient and practitioner expectations should only be measured before the start of treatment, as they will strongly be affected by the treatment itself. As well as notification on some items which were ambiguous and could be phrased clearer. For a summary of open comments from round two, see Table F.

# **Summary**

While participants rated that the majority of placebo features for the various types of placebo-controlled trials should be included in trial reports. There was an overarching theme from the comments that although this would be ideal, in practice this is not feasible, and it is more important to highlight where and how these features differ between the placebo and active interventions. In addition, as many of the features are already included within the TIDieR checklist, and TIDieR seems to be rarely used it may be more intuitive to create a separate simpler checklist/guide specifically for reporting the differences between placebo and active interventions.

**Table D. Ranking results**

| **Highest ranked (included)** | **Moderately ranked (undecided)** | **Lowest ranked (excluded)** |
| --- | --- | --- |
| **General** | **General** | **General** |
| Rationale for choice of placebo | Non-verbal cues |  |
| Similarities and differences to the active intervention | Duration of consultation prior to placebo treatment |  |
| Verbal information given to participants regarding placebo | Location of placebo treatment |  |
| Written information (e.g. information leaflet) given regarding placebo | Measured participant expectations/beliefs about the effectiveness of their allocated intervention (before, during and after)* |  |
| Duration of placebo treatment |  |  |
| Level of blinding | Measured practitioner expectations/beliefs about the effectiveness of the active and placebo/sham intervention (before, during and after)* |  |
| How blinding was attempted/achieved |  |  |
| Any modifications to placebo treatment |  |  |
| Planned adherence/fidelity assessment | How the placebo was marketed to those who allocated and/or delivered the intervention* |  |
| Actual adherence/fidelity |  |  |
| Any tailoring of placebo treatment |  |  |
| **Drug** | **Drug** | **Drug** |
| Placebo components/ingredients | Status of person administering the placebo treatment (e.g. the participant) | Status of person allocating the placebo treatment (e.g. the study nurse) |
| Appearance/colour |  |  |
| Dose | Size |  |
| Route of administration | Taste |  |
| Packaging | Manufacturer of the placebo* |  |
| **Acupuncture** | **Acupuncture** | **Acupuncture** |
| Type of sham acupuncture (e.g. Streitberger or Park needle (with description)? Needling in ‘wrong’ places? Not inserting needle? Toothpicks? Other?) |  |  |
| If and how sham acupuncture needle sensations were assessed |  |  |
| When did unblinding occur |  |  |
| Training of acupuncturist |  |  |
| **Surgery** | **Surgery** | **Surgery** |
| Similarities and differences to the ‘real’ post-surgery care and rehabilitation | Staff access to operation note detailing surgery (normally made available to staff providing post-operative care) |  |
| Depth/length of incisions | Training of surgeon |  |
| Local or general anesthetic |  |  |
| Participant access to operation note detailing surgery (normally made available to patients after their operation) |  |  |
| Post sham surgery care procedure |  |  |
| **Psychological/behavioural** | **Psychological/behavioural** | **Psychological/behavioural** |
| Training of practitioners to deliver the attention control |  |  |
| Practitioner's qualification |  |  |
| Practitioner's profession |  |  |
| Number of attention control sessions |  |  |
| Frequency of attention control sessions |  |  |
| What the practitioner does |  |  |
| What the practitioner says |  |  |
| What the participant is asked to do |  |  |
| Time from 1st attention control session to last |  |  |
| Any provision for participant-practitioner contact out of scheduled sessions |  |  |
| Homework expected of the attention control group participants in between sessions |  |  |

*Additional items suggested from round one

**Table E: Open comments and author’s note from Delphi Round one**

Below is a summary of the comments. We have reworded for simplicity, removed duplicates, and added some ‘Authors’ notes’ explaining what we have done to incorporate the comment.

General notes:

1. Some of the comments requested that we add items that already exist. For example, some requested that we add ‘duration’, when ‘how long?’ is a TIDieR item.
2. Many people promoted the need to measure patient and practitioner expectations. This could be a separate item, or it might be subsumed under blinding success. If expectations are different, then there will be no blinding. On the other hand, blinding is difficult to achieve in many interventions, so expectations might be better to measure than blinding. For now, we have included it as an additional item.

| **Comments on drug placebo** |
| --- |
| Comment: The important thing is to report *differences* between the ‘active’ and placebo/sham interventions. If this is done, then TIDieR-Placebo could be simplified. “E.g. I would be happy it the authors reported "The placebo tablet was indistinguishable from the genuine tablet" or words to that effect. Similarly, I assume that a placebo will be taken by same route and duration as real unless told otherwise. |
| *Authors’ note 1: This will be an important point to consider (whether to simplify TIDieR for placebo).* |
| *Authors’ note 2: Since we are looking for differences, the TIDieR-Placebo should mirror the standard TIDieR. We will ensure this point is taken forward in the eventual production of the placebo reporting guidance.* |
| Comment: Specify manufacturer of the placebo |
| *Authors’ note: This is useful, especially if it differs from the manufacture of the drug and we have added this to the TIDieR-Placebo items for drug placebos*. |
| Comment: Presumably, reporting the differences of the placebo to the active drug may release 'nocebo' effects. |
| *Authors’ note: TIDieR is about the reporting of the placebo in the trial report. It is related to, but distinct from the information shared with the patient. We will make a note of this comment and include it in the eventual publication.* |
| Comment: Expectations/beliefs of practitioners and patients (before, during, after) treatment are important. |
| *Authors’ note: For sake of completeness, we will add a general item: ‘measured participant expectations/beliefs about the effectiveness of their allocated intervention (before, during and after)’, and ‘measured practitioner expectations/beliefs about the effectiveness of the active and placebo/sham intervention (before, during and after)’. In our consensus meeting we will discuss whether this is subsumed under ‘blinding’. The effect of differing expectations could reasonably be assumed to be accounted for in the ‘success of blinding’ field (if there is lack of blinding, then there is the potential for differing expectations to influence results, and vice versa).* |
| Comment: Practitioner non-verbal cues and body language (eye contact, etc.) need to be reported |
| *Authors’ note: This is important. As far as the importance for placebo controls is concerned, the important thing is that it was the same in both groups. We will make a note in the eventual guidance that this kind of information should be included under ‘procedures’* |
| Comment: It is important to develop guidelines on this topic since manufacturers/etc. are not aware |
| *Authors’ note: We will do our best to share the results of this project widely so that it is taken up.* |
| Comment: 'Tailoring of placebo treatment' and 'modifications to placebo treatment' are too similar to be separate items. |
| *Authors’ note: We will consider combining these.* |
| Comment: Presentationally I think this detail would be much more clearly presented in a table than it would in narrative form e.g. under column headings such as "feature", "placebo", "intervention" so your eye can easily identify where the treatments deviate. For example, in this example it seems like info of minor interest that the nurse offers the placebo or active treatment. But it would seem more important if a nurse offered one and a clinician offered another. The info that might form the rows of the table appear to be the TIDier items? - They all seem to edge around the issue of "how similar or different were the intervention and placebo". Suggesting a really basic heading like this might be useful? |
| *Authors’ note: We think this is useful, a table with a column for ‘intervention’ and for ‘placebo/sham’. We will consider this in our eventual consensus meeting where the guidance is produced.* |
| **Comments on acupuncture placebo** |
| Comment: In the case of acupuncture, it should follow the STRICTA guidelines |
| *Authors’ note: The STRICTA guidelines are rather sparse about the description of sham acupuncture and note: ‘6a) Rationale for the control or comparator in the context of the research question, with sources that justify this choice 6b) Precise description of the control or comparator. If sham acupuncture or any other type of acupuncture-like control is used, provide details as for Items 1 to 3 above.’ TIDieR-Placebo might have to be adapted later as something like ‘STRICTA-Sham’.* |
| Comment: The participants' previous experience of acupuncture might be very important to identify whether they knew that the acupuncture was sham or not. |
| *Authors’ note: This is an important point, that we believe is included in the additional feature about expectations, which we have added.* |
| **Comments on surgery placebo** |
| No specific comments; folded into general comments and suggested additional elements. |
| **Comments on psychological/behavioural sham** |
| Comment: Therapists allegiance to the treatments how treatment vs. active control was "marketed" to therapists (e.g. "this is just to control for time" vs. "this controls for general factors of psychotherapy, which are powerful of itself") |
| *Authors’ note: We have added an additional item to the general TIDieR-Placebo items: ‘How the placebo was marketed to those who allocated and/or delivered the intervention (e.g. “this is just to control for noise/nonspecific factors” versus “this controls for general factors, which are powerful in and of themselves”)* |
| Comment: Yes, are the participants informed in advance of there being a "placebo" or not? If not the study is clearly deceptive, and thus it requires to address a different set of issues with respect to the mere presence of a "control" |
| *Authors’ note: There is an open and separate debate about informed consent and deception. We will note this in our eventual manuscript, however the relevance of this item is subsumed under ‘expectations’ and ‘blinding’ items.* |
| **Additional suggested features: general** |
| Proposed additional item: The way in which the assessment and information about the study was given (was it very matter of fact, or was there also interest in the person, some small talk etc). This can hugely influence how patients feel! |
| *Authors’ note: The existing TIDieR item states: ‘Procedures: Describe each of the procedures, activities, and/or processes used in the intervention, including any enabling or support activities. (NB: if available, simply cut and paste text from the paper).’ We will add a clarification that this also includes the type of communication between patients and practitioners.* |
| Comment: It is useful to report all the contextual factors presented in the therapeutic arena (e.g., features of clinicians, patients, treatment, patient-clinician relationship and healthcare setting). All the contextual factors should be reported independently from the nature of the different placebo treatment (e.g. sugar pill mimicking a drug; sham manual treatment mimicking a spinal manipulation) |
| *Authors’ note: This is important, and we believe that it is subsumed under ‘procedures’, ‘who’, ‘what’, ‘where’. We will discuss the importance of contextual factors as they relate to these in our manuscript. Certainly ‘differences in context factors’ should be reported, and we will make this clear.* |
| **Additional suggested features: drug** |
| Proposed additional item: It is also important to know what information patients receive about the randomization ratio of drug: placebo, e.g. if the ratio is 1:1 or 2:1 etc. Some meta-analyses showed that the likelihood to receive drug compared to placebo affects placebo effects (the higher the likelihood for the drug, the higher the placebo effect). |
| *Authors’ note: This will influence expectations and blinding, so is already subsumed within the blinding and expectations items.* |
| Proposed additional item: Side effects in placebo group expectations and or allocation guess of participants (several time points throughout the study) instructions to study personal how to interact with patients if the bring up sentiments of whether they believe to be on placebo vs. tx. (e.g. "I have side effect XY, I must be on active meds." How will study personal respond to that at follow up appointments) How was the study marketed (e.g. recruitment letter, referrals by GP) and whether that influences allocation beliefs (see new Cipriani Meta-analysis: Same Anti-depressant had different effects depending on whether it was supposed to be the "new drug" or the comparator; or see: https://www.frontiersin.org/articles/10.3389/fpsyt.2018.00424/full ) |
| *Authors’ note: This is subsumed within the additional ‘expectations’ item.* |
| Proposed additional item: I think the trustfulness of the person allocating the placebo treatment (e.g. study nurse) is also of importance. Trust in health care professionals might have a positive impact on subjective health (Birkhäuer et al., 2017) and may be of relevance for the placebo treatment. Thus, I would include this feature too (rate it as 'desirable'). |
| *Authors’ note: This is about the deliverer of the information and is included within the ‘WHO’ TIDieR item (‘For each category of intervention provider (e.g. psychologist, nursing assistant), describe their expertise, background and any specific training given.’)* |
| **Additional suggested features: acupuncture** |
| No specific comments |
| **Additional suggested features: surgery** |
| Proposed additional feature: It is important to report what constitutes the "critical surgical element" that has been removed from the active operation to inform the placebo - given that surgical placebos are invasive, it is important to report what measures were taken to minimise the potential harm of the placebo - although not directly associated with the features of the placebo itself, it would be important to report any additional measures taken to enhance the informed consent process given that surgical placebos are invasive/ |
| *Authors notes: This is important conceptually. However, with a complete description that includes all differences, the ‘critical surgical element’ will be implied. Likewise, with a potential harm, if the interventions are well described, then the harms can be critically appraised by an independent reader.* |
| **Additional suggested features: behavioural/psychological** |
| Potential additional item: Yes, are the participant informed in advance of there being a "placebo" or not? If not, the study is clearly deceptive, and thus it requires to address a different set of issues with respect to the mere presence of a "control" |
| *Authors’ note: This is about informed consent rather than description of the intervention.* |
| **Other comments** |
| Comment: Everything is important in generating positive health outcomes, with real or inert treatments, starting with the parking lot attendant. The patient is referred by his/her GP. How enthusiastic is s/he about the treatment under study? If the referring doc is bubbling over with enthusiasm for the new treatment, pts will do better regardless of the content of their treatment. |
| *Authors’ note: This is about similarities and differences, which we agree will be considered implicitly and explicitly.* |

**Table F: Open comments from Delphi Round two**

| **Comments on highest ranked placebo features** |
| --- |
| **General item: similarities and differences to the active intervention** |
| Comment: “… I see it as more of a summary of other more specific TIDieR items. If it's not specific enough, then it won't be a useful item to include. Perhaps it should be worded as something like "consider providing a summary of the similarities and differences of the placebo to the active intervention". It also overlaps with items on the more specific lists presented above…The final list should be as relevant to all placebo interventions as possible, but not clunky/overly long with all other types of interventions with their 'own lists'” |
| Comment: “…might need unpacking and seems to summarise pretty much everything we would want to capture, not sure what it adds without listing the various aspects that we would want to know about in terms of similarities/differences. Consider something more like "Any other similarities or differences to the active intervention that are not captured above" as the final item in the checklist.” |
| **General item: Any modifications to placebo treatment** |
| Comment: “Would be better phrased Any modifications (e.g. tailoring, titrating) to placebo treatment (if you intend to get at modifications for the individual patient - or is the intention to get at modifications for the whole placebo group across the board, in which case this needs to be clearer).” |
| **Drug specific item: Placebo components/ingredients** |
| Comment: “…is ambiguous. If it is specifically for placebo drugs only, then ingredients would seem to be the most appropriate term. 'Components' encourages people to think of contextual components, which warrant their own item and are relevant to all placebos, not just drug placebos.” |
| **Acupuncture specific item: When did unblinding occur** |
| Comment: “I consider it problematic that unblinding is an issue for acupuncture, but not for other treatments. Obviously, unblinding can happen in case of all treatments in different ways, and when it occurs it can (but not necessarily does) cause bias. Furthermore, assessing unblinding is problematic in itself, as perceived treatment effects can interact with guesses/assessments (therefore, unblinding is not an intem in CONSORT). If it is inlcuded for acupuncture but not for other treatments, there must be a rationale. Anyhow, I wonder whether the issue of unblinding should be discussed in a more open manner in the background paper of the recommendations than being a recommendation. What exactly should be recommended: Report a) whether you used a test of unblinding, b) the rationale for doing so, c) the findings...?” |
| **Surgery specific item: Depth/length of incisions** |
| Comment: “I don't think that "Depth/length" of incisions is any relevant or reliable. Surgeons typically do NOT consider that parameter key component as individual variability is common.” |
| **Psychological/behavioural specific item: Homework expected in between sessions** |
| Comment: “is very CBT esque but might not fit so well with other types of psych/behavioural interventions. Maybe 'activities' expected of the control group between sessions?” |
| **Psychological/behavioural specific example: Time from 1^st^ attention control session to last** |
| Comment: “this needs to be broader than just this aspect of time, but all elements such as length of each session, scheduling of sessions, total time, etc (as per original TIDieR item)” |
| **Comments on moderately ranked placebo features** |
| **General item: Measure participant/practitioner expectations/beliefs about the effectiveness of their allocation intervention (before, during and after)** |
| Comment: “Expectation can be interpreted as being a very conscious, deliberative process, but some aspects of prior learning/conditioning regarding placebos may not be conscious (e.g. Stewart-Williams and Podd, 2004). If the expectation item is included to cover conditioning and the effects of prior experience perhaps this should be highlighted.” |
| Comment: “Very important to measure expectations, so having an item about reporting on this could encourage more triallists to measure expectations. However, not sure we need to be measuring expectations before during and after, this seems like a high burden for participants and for triallists and the act of repeated measures may itself shape expectations. Suggest it is most important to measure expectations before administering the intervention but after informing the patient about it.” |
| Comment: “Measuring expectations and beliefs after the start of treatment is problematic, as they will be strongly affected by the treatment itself, e.g. whether the treatment is experienced to be working or not. So expectations will rise sharply for example if there is an immediate improvement in symptoms related to the treatment. So suggest measure expectations and beliefs only before treatment has commenced, and explain how these factors are to be used in the trial, for example they can be used as covariates in the analysis.” |
| Comment: “Measuring blinding success would capture all the 'measure expectations' fields.” |
| **General item: How the placebo was marketed to those who allocated and/or delivered the intervention** |
| Comment: “How robust an answer do you get to people's mindset about placebo/neutrality to the RQ. Similarly I think the sell could vary daily and could even be inconsistent within people.”  Drug specific item: Status of person administering the placebo treatment (e.g. participant) |
| Comment: “the word "status" is confusing. Presumably this means something about the role of the person in delivering the intervention or some such. People's responses to this question may be influenced by how they have interpreted what is meant by 'status” |
| **Drug specific item: Size** |
| Comment: “"size" is an odd word to use here - for drug-specific placebo, this needs to be about dose” |
| **Drug specific item: Manufacturer of the placebo** |
| Comment: “I'm not sure to what extent this would be useful, primairly beciase I don't knwo how I woudl interpret this information. Might be enough just to report whether the same manufacturer was used for the placebo and the drug.” |
| **General comments** |
| **Drug** |
| Comment: “In case questions regarding shape, size, taste etc means ”in relation to the real drug” it is of course essential, it has to be indistinguishable from verum. The general choice of color, shape etc in a study is of minor importance.” |
| Comment: “The physical characteristics of drugs and placebos have been shown to be significant many times. E.g., tablet vs. capsule, colors, shapes, form, number.” |
| **Acupuncture** |
| Comment: “2.) Regarding the comment, “The STRICTA guidelines are rather sparse about the description of sham acupuncture”, I do not agree. (I need to declare I have been involved in developing STRICTA). Items 2 to 3 in STRICTA, when used to describe the sham control intervention, need to cover the following items where relevant: Details of (sham) needling: 2a) Number of needle insertions per subject per session (mean and range where relevant) 2b) Names (or location if no standard name) of points used (uni/bilateral) 2c) Depth of insertion, based on a specified unit of measurement, or on a particular tissue level 2d) Response sought (e.g. de qi or muscle twitch response) 2e) Needle stimulation (e.g. manual, electrical) 2f) Needle retention time 2g) Needle type (diameter, length, and manufacturer or material). (Sham) Treatment Regimen: 3a) Number of treatment sessions 3b) Frequency and duration of treatment sessions. STRICTA is within the CONSORT family of reporting guidelines, and so the TiDieR reporting should not specify anything less than that identified as important by acupuncturist triallists and specialists and methodological experts for STRICTA. This is important for the field, as we have had enough of over-simplified reporting generally, and would not want such simplifications to be supported by TiDieR.”  Comment: “3.) The problem with using percentages to establish whether an item should be in the TiDier scheme or not, is that many participants have no experience of sham acupuncture trials, or the acupuncture field generally, so may happily agree with suggestions, and in doing so overweight opinion towards a consensus that does not reflect the expertise in the acupuncture field. This is a worrisome methodological weakness in TidieR.” |
| Comment: “I understand the plan for placebo features achieving consensus (ranked as "essential" by ≥70% participants or "essential or desirable" by ≥85%) will be incoorporated into the TIDieR - Placebo extension, "unless any significant objection is received". I do have an objection, because many participants from outside the acupuncture field appear to be supporting an over-simplified level of reporting. Given the current role of STRICTA, which is within the CONSORT family of reporting guidelines, is there some way that TiDier reporting does not reduce the recommended levels of reporting of acupuncture trials as set out in STRICTA?” |
| **Psychological/behavioural** |
| Comment: “I'm not sure you can have a psychological/behavioural placebo. See Irving Kirsch's paper: Placebo psychotherapy: synonym or oxymoron? <https://www.ncbi.nlm.nih.gov/pubmed/15827992>” |
| **All types of placebo control** |
| Comment: “What the practitioner "Knows." Always remember the Gracely study.” |
| Comment: “General Is 'active intervention' the clearest term given that 1) it is presumably being evaluated for efficacy and 2) placebos might be 'active'? Is there a way of reframing as simply intervention vs placebo control condition? This is a side issue from your project but adverse effects of placebos are often quite poorly evaluated and reported (especially for psychological/behavioural interventions). It is strictly speaking covered by the outcomes section of CONSORT but the CONSORT statement doesn't specify that all outcomes should be reported for placebo groups or highlight that outcomes include adverse effects. It seems like a missed opportunity not to highlight in your statement.” |
| Comment: “I can TOTALLY see why we would like to see this level of detail however I do have major concerns about a) authors willingness to do this and probably their concern about using up many words doing so. b) reviewers or editors appetite for checking each of these things off. I think with such a long list the only way I can imagine this working is in a table. and the answer is very short and the table avoids duplication. As a journal ed there are several of these which are not intuitive and involve you knowing quite a bit about placebo research (which you may well not). So wherever possible I would use common language rather than research jargon to get the info you want. I would have no idea what I should see next to the following requests (unless plainer English is used) - Attention control - Any modifications to placebo treatment - Planned adherence/fidelity assessment - Actual adherence/fidelity” |
| Comment: “… a number of details depend on the aim of the study (if it is experiemntal placebo research usually morer detail is needed, while in some other studies statements such as "active drugs and placebos capsules were indistinguishable in size, appearance and taste, and packaged in identical, consecutively numbered containers"; taste is a difficult thing and asking to report it might induce cheating: the capsules might taste identical but if you open them...)” |
| Comment: “Presumably there are various motivations for including this detail 1) appraise whether it was a fair test, 2) details to allow you to replicate either for further research or in practice. To me the main job of the paper reporting a trial is the first one. But people doing SR or looking for implementation may need the second lot of info? I really keep coming back to the same thought that perhaps the detail should be on a table or even tick box exercise with a short summary in the paper of in what important ways did the placebo differ from the other treatment. And what are the magic ingredients of the placebo. I guess this could serve a bit like a table 1 of baseline characteristics. But instead be a table which makes it easy to appreciate if the placebo differed in notable ways. My inclination is that all of the details are important IF they differ. But if they don't differ you need a very quick and easy way to be feel reassured. In my experience this is very rarely found in text.” |
| Comment: “In specific field of healthcare (e.g. physiotherapy, chiropractic) is impossible to adopt a "real" therapy versus a "sham" therapy (e.g. real spinal manipulation VS sham manipulation). Therefore there is a need: 1) to explicit which elements describe "real" and "sham" therapy; 2) to control for confounders between groups (e.g. contextual factors). Rossettini G, Testa M.Manual therapy RCTs: should we control placebo in placebo control? Eur J Phys Rehabil Med. 2018 Jun;54(3):500-501. doi: 10.23736/S1973-9087.17.05024-9 Maddocks M, Kerry R, Turner A, Howick J. Problematic placebos in physical therapy trials.J Eval Clin Pract. 2016 Aug;22(4):598-602. doi: 10.1111/jep.12582” |
| Comment: “I think some guidance for the reporting on contextual factors would be very helpful - whether this should take the form of multiple single items (which seems to be the approach in the current draft), or one overarching item that cues people to report on multiple aspects, I think could be discussed. It would be helpful to draw on relevant theory/frameworks here. Options would include the 5 contextual domains described in Di Blasi's Lancet paper, or the more recent work on context (but in a public health context) by the CIHR/NIHR - <https://www.ncbi.nlm.nih.gov/books/NBK498645/>” |
| Comment: “…there might be some useful Qs to see answered in a more narrative way. eg what was tough about making the placebo and the other intervention/s the same? How did you do it? Using the ticklist table summarise the ways in which the placebo might have differed?” |

**References**

1. Hoffmann TC, Glasziou PP, Boutron I, Milne R, Perera R, Moher D, et al. Better reporting of interventions: template for intervention description and replication (TIDieR) checklist and guide. BMJ : British Medical Journal. 2014;348:g1687. doi: 10.1136/bmj.g1687.

2. Webster RK, Howick J, Hoffmann T, Macdonald H, Collins GS, Rees JL, et al. Inadequate description of placebo and sham controls in a review of recent trials. European journal of clinical investigation. 2019:e13169.

3. Bello S, Wei M, Hilden J, Hróbjartsson A. The matching quality of experimental and control interventions in blinded pharmacological randomised clinical trials: a methodological systematic review. BMC Medical Research Methodology. 2016;16(1):18. doi: 10.1186/s12874-016-0111-9.

4. Howick J, Hoffmann T. How placebo characteristics can influence estimates of intervention effects in trials. Canadian Medical Association Journal. 2018;190(30):E908-E11. doi: 10.1503/cmaj.171400.

5. Moher D, Schulz KF, Simera I, Altman DG. Guidance for developers of health research reporting guidelines. PLoS medicine. 2010;7(2):e1000217. Epub 2010/02/20. doi: 10.1371/journal.pmed.1000217. PubMed PMID: 20169112; PubMed Central PMCID: PMCPMC2821895.

6. Murphy M. Consensus development methods, and their use in clinical guideline development. Health technology assessment. 1998;2(3):1-88.
