# Supplementary material for: TIDieR-Placebo: A guide and checklist for reporting placebo and sham controls
Source: PLoS Med. 2020 Sep 21;17(9):e1003294. doi: 10.1371/journal.pmed.1003294 (PMC7505446; doi:10.1371/journal.pmed.1003294)
Supplement: S3 Text — (DOCX) [file pmed.1003294.s006.docx]

# **S3 Text. Additional Results**

## **Additional Items Discussed at Consensus Meeting**

The other additions/modifications to the original TIDieR involved elaboration, to reflect evidence about placebo-related effects (for example, body language of healthcare practitioner). The group found that these items could be collapsed into existing TIDieR items, provided that some additional explanation was provided. Specifically, additional items suggested by Delphi respondents such as measuring patient and healthcare practitioner expectations before and after treatment, and how the intervention(s) were marketed could be accounted for by measuring the success of blinding.

In fact, operationally testing the success of blinding and measuring expectations are the same, as the success of blinding is measured by asking trial participants what treatments they expect to have received. The need to determine when participant and staff access to the operation notes in a sham surgery trial was deemed important and also accounted for by measuring the success of blinding.

Some broader issues were also raised, about the rationale for conducting a placebo-controlled trial in the first place was important. If a different design would have been preferable, then the question of whether the placebo or sham control was adequately described was moot. However, it was agreed that this was not a property of the placebo or sham intervention, and that it should be explored in a separate paper authored by a subset of members from the group.

We also discussed the importance of reporting control interventions in cessation trials. Probably too complex for this more general paper, but a point I made was that in drug cessation trials, drug cessation is the active intervention to be tested. So, the appropriate placebo comparator is not, in contrast to what is commonly practiced, introducing fake medication, as this this would only evaluate the effect of pharmacological withdrawal while continuing the act of taking pills. For evaluating the total clinical effect of drug cessation the comparator should be ‘placebo cessation’: the visible act of cessation while in fact the pharmacological intervention is invisibly continued. This requires a sophisticated approach in which the medication is administered unnoticed, for example, via other, not deprescribed medication, or food. While, as far as we know, this design has not been applied yet, it is good to realize what the appropriate comparator would really be [1].

**References**

1. Knottnerus JA, Tugwell P. Inadequate comparators produce misleading results - the importance of good comparison practice. J Clin Epidemiol. 2019;110:v-vi. Epub 2019/05/20. doi: 10.1016/j.jclinepi.2019.04.010. PubMed PMID: 31103071.
